# Barcoding *Eophila crodabepis* sp. nov. (Annelida, Oligochaeta, Lumbricidae), a Large Stripy Earthworm from Alpine Foothills of Northeastern Italy Similar to *Eophila tellinii* (Rosa, 1888)

**DOI:** 10.1371/journal.pone.0151799

**Published:** 2016-03-28

**Authors:** Maurizio G. Paoletti, Robert J. Blakemore, Csaba Csuzdi, Luca Dorigo, Angelo Leandro Dreon, Federico Gavinelli, Francesca Lazzarini, Nicola Manno, Enzo Moretto, David Porco, Enrico Ruzzier, Vladimiro Toniello, Andrea Squartini, Giuseppe Concheri, Marina Zanardo, Javier Alba-Tercedor

**Affiliations:** 1 Università degli Studi di Padova, Dipartimento di Biologia, Padova, Italia; 2 Hanyang University, College of Natural Science, Seoul, Korea; 3 Eszterházy Károly College, Department of Zoology, Eger, Hungary; 4 Museo Friulano di Storia Naturale, Udine, Italia; 5 ESAPOLIS, Padova, Italia; 6 Université de Rouen, Laboratoire ECODIV, Mont Saint Aignan Cedex, France; 7 Natural History Museum, Department of Life Science, London, United Kingdom; 8 Federazione Speleologica Veneta-G.S. C.A.I., Laboratorio di Biospeleologia di Villa Papadopoli, Vittorio Veneto, Italia; 9 Università degli Studi di Padova, Dipartimento di Agronomia Animali Alimenti Risorse Naturali e Ambiente, DAFNAE, Padova, Italia; 10 Universidad de Granada, Departamento de Zoología, Facultad de Ciencias, Granada, España; Queensland University of Technology, AUSTRALIA

## Abstract

A new Italian earthworm morphologically close to the similarly large and anecic *Eophila tellinii* (Rosa, 1888) is described. Distribution of *Eophila crodabepis* sp. nov. extends over 750 km^2^ from East to West on the Asiago Plateau and Vittorio Veneto Hills, from North to South on mounts Belluno Prealps (Praderadego and Cesen), Asiago, Grappa and onto the Montello foothills. This range abuts that of *Eophila tellinii* in northern Friuli Venezia Giulia region. Known localities of both *E*. *tellinii* and *E*.*crodabepis* sp. nov. are mapped. mtDNA barcoding definitively separates the new western species from classical *Eophila tellinii* (Rosa, 1888).

## Introduction

Study of megadrile earthworms is easily justified due to their key ecological role as drivers of soil formation in association with microorganisms (especially bacteria and fungi) [1,2,3,4].

In 1888 Daniele Rosa described *Allolobophora tellinii* (now *Eophila tellinii*) the largest Italian earthworm (up to 800 mm according to Paoletti [5,6]), characterized by a livery of puce and purple bands in the middle of each segment. It was included in the “Classical” taxonomic systems of Michaelsen [7] and Stephenson [8] (under genus *Helodrilus* Hoffmeister, 1845). Its ecological category is anecic or deep-burrowing (cf. [2,5,9,10,11] with vertical burrows going meters deep although feeding is mostly on decaying litter on the soil surface, especially at night or during rain. *E*. *tellinii* is often located at the base or under large rocks or in the roots of trees with a marked preference for hazel (*Corylus avellana*, L.) and decidous forest [12]. Such microhabitats ensure a greater protection from predators and sudden changes of temperature and humidity [3].

*E*. *tellinii* localities are characterized by mull calcareous grey soils, sometimes stony and with rock outcrops; the species has an altitudinal range between 100–1,200 m. This species colonize the Southern Prealpine slopes and hills covered by deciduous woodlands [5,13,14,15]. *Eophila* predators include the badger *Meles meles* (Linnaeus, 1758), carabid beetles *Abax parallelepipedus* (Piller & Mitterpacher, 1783) and *Carabus* (*Procerus*) *gigas* (Creutzer, 1799), *Eupolybothrus grossipes* (C.L. Koch, 1847), *Xerobdella sp*. (von Frauenfeld, 1868) that share the same range [5].

Despite their ecological importance, knowledge of earthworm taxonomy and ecology is remarkably limited in Italy as elsewhere and the specific roles of earthworms in soil formation in rural environments—especially in vineyards but in forests as well—is largely underestimated. The large and charismatically coloured *E*. *tellinii* exemplifies this: in the current study “*E*. *tellinii*” is found to actually comprise two taxa separable on morphology as well as on genetics and distribution pattern.

## Materials and Methods

### Morphology

Earthworms were collected at different stations both by spade-fork digging and expulsion using 0.2–0.5% formaldehyde [16] or mustard powder (25 g/l) [17]. Specimens were preserved in 80% ethanol then stored at +4°C and most are kept in the Biology Department of the University of Padua, Via Ugo Bassi 58b, 35121 Padova (Italy) although some were transferred to other institutions as noted under species’ description. Three earthworms (*Crevada 6*, *Clauzetto 2*, *Ragogna 2*) are deposited in the Department of Zoology of the University of Granada (Spain) and six (*Grappa Mount 2*, *Ragogna 3*, *Ragogna 1*, *Val Posan 2*, *HNHM 6899*, *HNHM 12678*) in the Hungarian Natural History Museum, Budapest. The specimen called “*Campo Solagna 17*” was subjected DNA-barcoding and not kept. Two specimens (*Ragogna 1*, *Grappa Mount 2*) were subjected to anatomic dissection to observe the internal features. A specimen (*HNHM 6899*) was bisected and the middle part sectioned to observe musculature. Three specimens (*Crevada 6*, *Clauzetto 2* and *Ragogna 2*) were sent to Professor Javier Alba-Tercedor of the University of Granada for micro-tomographic scanning with a Bruker-Skyscan 1172. This technique examined features of specimens without dissection, in particular the intestinal typhlosole shape and extent.

LOMBRI software [18] was used for identification confirmed by scientific literature (listed in synonymy) using family and species systematics of Blakemore [19,20].

### Ethics Statement

The earthworm samples were collected in public areas in the provinces of Udine, Treviso and Vicenza on forested areas with no special requirements needed for collection permits. No endangered or protected species were involved.

### DNA barcoding

#### Sampling

**Fig 1:**
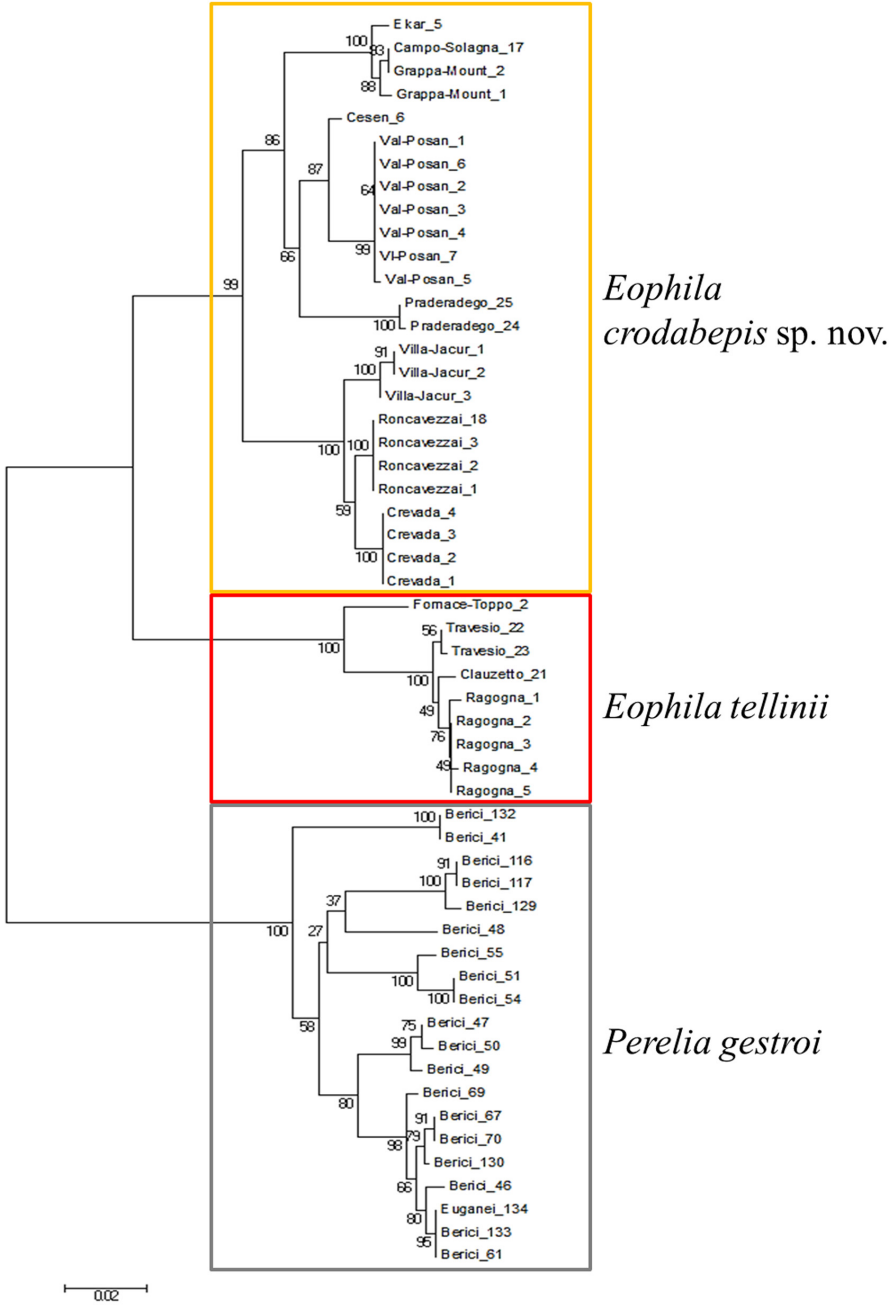
Cluster of the sequences from *E*. *tellinii* and *E*. *crodabepis* sp. nov sampled in the studied area with *Perelia gestroi* shown for comparison.

Nine specimens of *E*. *tellinii* and 25 of *E*. *crodabepis* sp. nov. were sequenced. In order to have a comparison point for specific divergence, 20 specimens of *Perelia gestroi* (Cognetti 1905) were also sequenced (Fig 1). Uncertainty of latter taxon name and position detailed in Blakemore [20].

#### Sequencing

Specimens were sequenced for mtDNA-barcoding region (658bp of the mitochondrial cytochrome oxidase subunit I 5’ end [21]).

DNA was extracted from one mm^3^ of muscle taken from the ‘tail’ of each specimen and preserved in 98% ethanol. The extraction took place following the standard Canadian Center for DNA Barcoding (CCDB) automated protocol [22] using 96-well glass fibre plates [23]. Amplification used M13 tailed primers (C_LepFolF/C_LepFolR) and followed standard CCDB protocol for PCR reactions [24] with end products checked on a 2% E-gel 96Agarose (Invitrogen). Unpurified PCR amplicons were sequenced in both directions using M13 tailed primers, their products subsequently purified using Agencourt CleanSEQ protocol and processed using BigDye version 3.1 on an ABI 3730 DNA Analyzer (Applied Biosystems). Sequences were assembled and edited with Sequencher 4.5 (GeneCode Corporation, Ann Arbor, MI, USA). Alignments used BIOEDIT version 7.0.5.3 [25]. Sequences are publicly available on GenBank (KT352925-KT352978) and on BOLD in the dataset [DS-NEO1] through the following DOI: dx.doi.org/10.5883/DS-NEO1.

#### Data analysis

Distance analyses were performed with MEGA6 [26], using a Neighbor-Joining [27] algorithm with the Kimura-2 parameter model [28] to estimate genetic distances. The robustness of nodes was evaluated through bootstrap re-analysis of 1000 pseudoreplicates. Molecular Operational Taxonomic Units (MOTUs) were defined with the software ‘mothur’ [29].

### Nomenclatural Acts

The electronic edition of this article conforms to the requirements of the amended International Code of Zoological Nomenclature, and hence the new names contained herein are available under that Code from the electronic edition of this article. This published work and the nomenclatural acts it contains have been registered in ZooBank, the online registration system for the ICZN. The ZooBank LSIDs (Life Science Identifiers) can be resolved and the associated information viewed through any standard web browser by appending the LSID to the prefix “http://zoobank.org/”. The LSID for this publication is: ***Eophila crodabepis* Paoletti sp. nov.**

**urn:lsid:zoobank.org:pub:53662919-7E2D-4DC6-BB89-C60D2FC6C193**

The electronic edition of this work was published in a journal with an ISSN, and has been archived and is available from the following digital repositories: **PubMed Central**.

## Results

### Taxonomy

***Eophila tellinii* (Rosa, 1888).**

(S1 and S2 Figs)

*Allolobophora tellinii* Rosa, 1888: 1. Type locality northeast of Italy, Ragogna hills in the province of Udine (Friuli Venezia Giulia). Syntypes in O1579 ITALIA, Friuli, Ragogna (UD) Torino (Turin) Regional Museum of Natural Science (not examined) [30].

*Allolobophora* (*Eophila*) *tellinii*: [31]: 10; [32]: 93.

*Helodrilus (Helodrilus) tellinii* [7]: 500.

*Eophila tellinii*: [8,14,33,34,35,36]: 73, figs 1 and 2 (misdated “Rosa, 1894”); [37,38]: 481 (misdated “Rosa, 1886”); [11].

**Fig 2:**
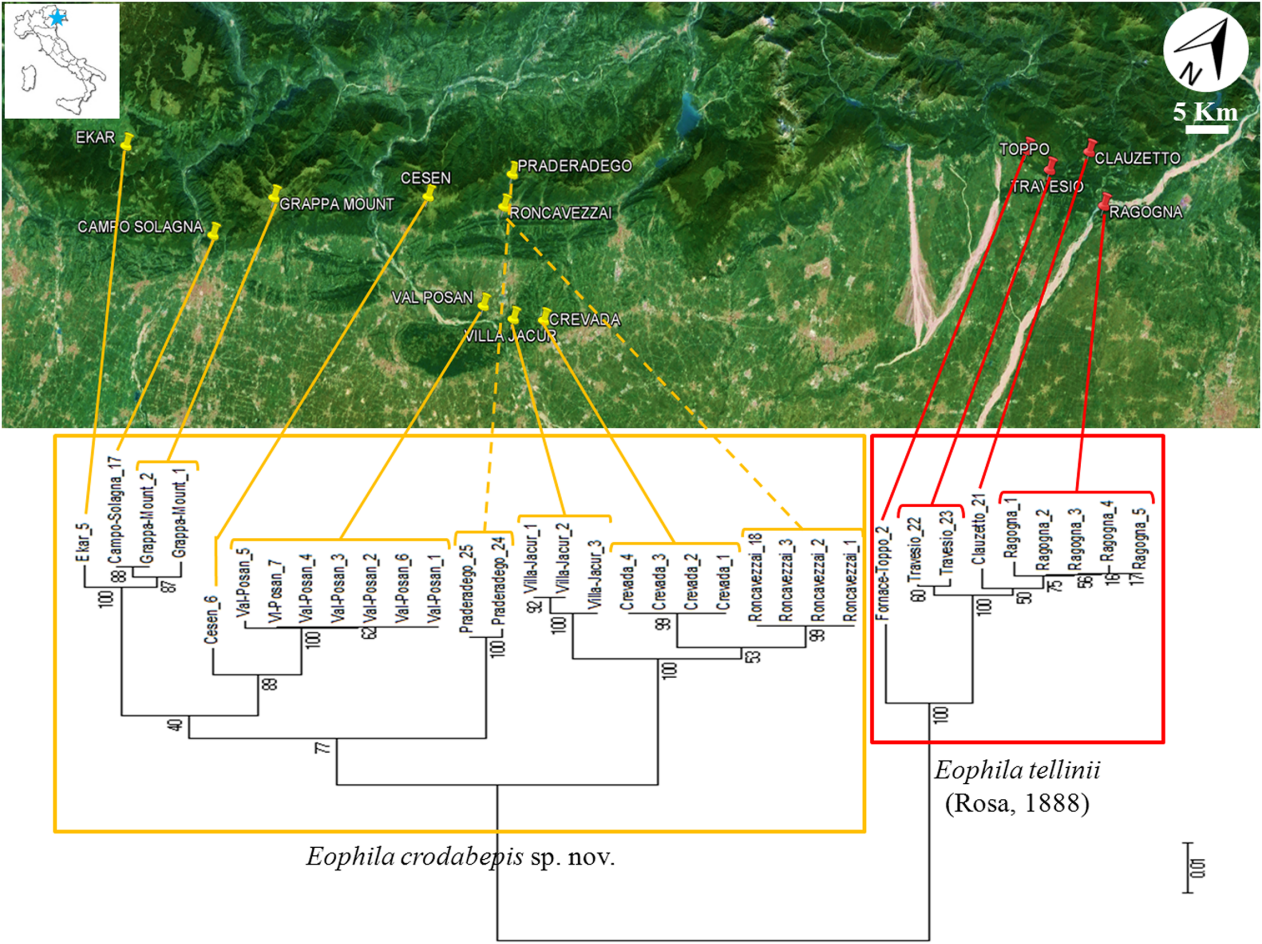
Barcoding Cluster of *E*. *crodabepis* sp. nov. (in yellow) in relationship with *E*. *tellinii* (in red) corresponding to their geographical distributions.

Rosa [30] said: “*Questo lombrico*, *di grandissime dimensioni*, *me è noto per un solo esemplare raccolto del signor Achille Tellini laureando in scienze naturali a Ragogna nel Friuli (alt*. *220 m) sul finire dello scorso aprile*”, i.e., he had a single, large specimen collected in April from Ragogna nel Friuli (= *Ragogna*) at 220 m of altitude.

Newly collected specimens are from several locations in Friuli Venezia Giulia (S1 Table).

Fig 2 shows geographical distribution of the samples integrated with their barcode clusters.

Other data in the literature originate from specimens deposited in the Museo Civico di Zoologia, Roma (Italy) [14] some of which are now missing (S2 Table).

Fixed adult lengths 170–360 mm (syntype 300 mm). First dorsal pore 5/6. Segments 250–341 in adult specimens (syntype 264) and from 193–275 in the immature specimens (including specimens which have probably been victims of predation). Color dark puce with purple bands in the middle of each segment; sometimes bands are less visible on the terminal portion of the body or pigment is lacking; intersegments are always pale. Adult specimens weighed 11.4–28.2 g. Maximum width at clitellum 10–14 mm. Clitellar height 9–11 mm. Length of living specimens can be 600–800 mm. Prostomium epilobous. Setae closely paired. Clitellum 26,27,28–40,41,42,44 involving fourteen to eighteen or nineteen segments. Tubercula pubertatis 30,31,32–37,38,39,40 (syntype 32–37). Spermathecal pores paired in 9/10 & 10/11 in *cd*. Female pores on segment 14 above setae *b*. Male pores on segment 15 between setae *b* and *c*, with or without small sized tumescences confined to segment 15. Setal papillae in adults on some segments of 6–13, 7–12, 7–13, 8–11 or 8–12. Body shape cylindrical depressed caudally. Setal ratio on segment 12 in adult specimens: *aa*: 5–11; *ab*: 1; *bc*: 4–6; *cd*: 0.6–0.9; *dd*: 26.7–42; *U* (circumference): 33–54; mean: *aa*: 8.9; *ab*: 1; *bc*: 5; *cd*: 0.8; *dd*: 33.7; U: 42.7 (S3 Table and Table 1). Nephridial pores irregularly alternate between setal lines *b* and well above *d*.

**Table 1:** Setal ratio of *Eophila tellinii* on 12th segment.

SAMPLE	*aa*	*ab*	*bc*	*cd*	*dd*	*U*
***Clauzetto 21***	9.5	1	5.5	0.8	42.0	38.0
***Ciaurlec Mount***	8.0	1	5.0	0.8	38.0	35.0
***Ragogna 1***	11.0	1	5.3	0.7	26.7	51.7
***Ragogna 3***	11.0	1	5.4	0.6	29.0	54.0
***Ragogna 4***	5.0	1	6.0	0.9	36.0	33.0
***Travesio 22***	10.0	1	4.0	0.8	34.0	42.0
***Travesio 23***	8.0	1	4.0	0.7	30.0	45.0
**MEAN**	**8.9**	**1**	**5.0**	**0.8**	**33.7**	**42.7**

Septa 5/6–7/8, 12/13–14/15 thickened, 8/9–11/12 strongly strengthened. Excretory system holoic, first pair of nephridia in 4. Nephridial bladders from 6 proclinate J-shaped, after the clitellum almost U-shaped. Hearts 6–11 with a pair of extraoesophageal vessels in 12. Calciferous glands in 10–12 with large vertical diverticula in 10. Crop large in 15–16 and muscular gizzard in 17–19. Typhlosole large, trifid, begins around segments 23–45 and ends variably before pygidium. Testes and male funnels in 10 & 11 seemingly free. Vesicles four pairs in 9–12, the first two pairs quite small and easy to overlook, those in 11 & 12 large. Spermathecae globular in 10 & 11. Ovaries moderate, pear-shaped in 13, ovaric sac moderate in 14 pendant from septum 13/14. (S3 Table).

Up to a dozen *E*. *tellinii* specimens per square meter were collected from Friuli Venezia Giulia, found either alone or with other species of earthworms (S1 Table). Associates were deep-burrowing *Octodrilus complanatus* (Dugés, 1828) and *Octodrilus pseudocomplanatus* (Omodeo, 1962) and other species belonging to different ecological categories (i.e., litter species, topsoil and/or subsoil species) such as *Octodrilus lissaensis* (Michaelsen, 1891), *Octodriloides phaenohemiandrus* (Zicsi, 1971) and *Octolasion lacteum* (Örley, 1881).

In the laboratory, *E*. *tellinii* can live under water for at least 3–4 weeks, this possibly linked to its particular hemoglobin [39]. Sometimes under very wet field conditions, *E*. *tellinii* and *E*. *crodabepis* sp. nov. have been found moving on the soil surface, earning them a local name of “vier de la pluje” or worm of the rain in Carnia region of Friuli Venezia Giulia.

Our new specimens comply within acceptable limits of the original description of *E*. *tellinii* except in the number of seminal vesicles which was stated as two pairs in segments 11 & 12 by Rosa [30] compared to four pairs found in 9–12. However, in our specimens the first two pairs in 9 & 10 are small and easily overlooked which might be the reason why Rosa missed them.

***Eophila crodabepis* Paoletti, 2016 sp. nov.**

*Eophila crodabepis* Paoletti sp. nov.

urn:lsid:zoobank.org:pub:53662919-7E2D-4DC6-BB89-C60D2FC6C193

**Fig 3:**
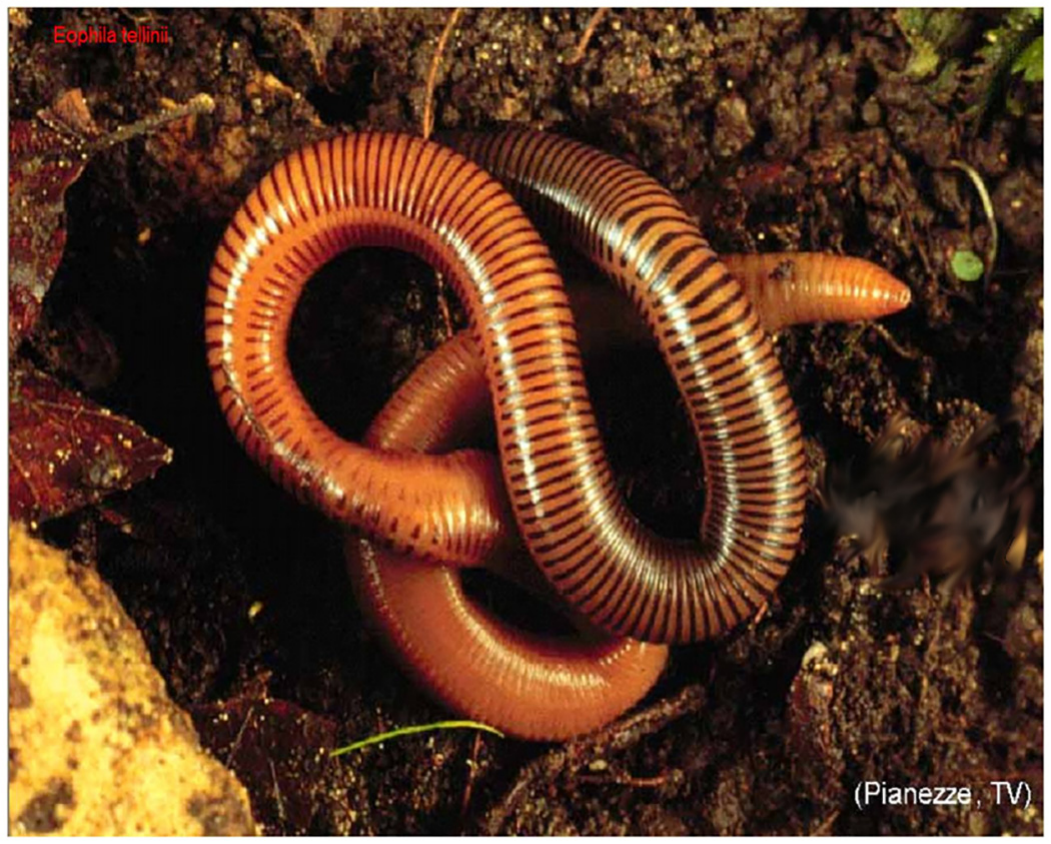
*Eophila crodabepis* sp. nov. specimen found upon the litter layer.

**Fig 4:**
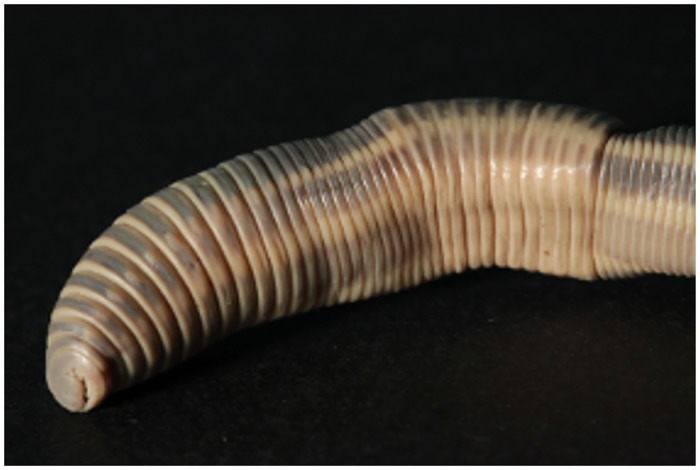
Holotype of *Eophila crodabepis* sp. nov. (*Crevada 3* specimen).

Figs 3 and 4; S3 Fig.

Specimens studied were collected in different localities in Veneto region (Table 1)

Holotype: *Crevada 3* [BOLD sampleID = IT_MGP_Crevada_3,

Genbank accession = KT352951], in the Verona Natural History Museum.

Paratypes: *Crevada 1* (P1), *Crevada 2* (P2), *Crevada 4* (P3) University of Padua, HNHM 6899 (P4), *HNHM 12678* (P5) deposited in the Hungarian Natural History Museum, Budapest.

The name of the new species is taken from an acronym dedicated to Giovanni **C**anestrini, Daniele **Ro**sa, Charles **Da**rwin, Antonio **Be**rlese, **Pi**etro Omodeo and Filippo **S**ilvestri, for various reasons relating to their scientific contributions to soil biology and earthworm studies in Italy and further abroad. The new taxon may be cited as “*Eophila crodabepis* Paoletti, 2016 in Paoletti et al. 2016”.

Body cylindrical, caudally depressed. Adult length (fixed): 100–240 mm by 10 mm diameter (holotype 130 mm, P1 120 mm, P2 110 mm, P3 140 mm, P5 240). Adult living specimens length can be 400–600 mm.

Colour: Purplish-brown bands in the middle of each segment with puce intersegments; bands are less visible or absent caudally in some specimens, or pigment is lacking from posterior half of ventral side or form ventral and lateral sides or entirely lacking ventrally; it is always lacking along setal lines *cd*. Adult specimens weigh 2.58–12.5 g (holotype 5.07 g, P1 2.64 g, P2 2.58 g, P3 4.927 g). Fixed specimens diameter at the clitellum: from 6–12 mm (holotype 10 mm, P1 and P2 8 mm, P3 9 mm, P5 11 mm). Fixed specimens height at the clitellum 6–10 mm (holotype 9 mm, P1 6 mm, P2 6 mm, P3 8 mm). Total segments 139–260 in adult specimens and 132–267 in immatures (including probable predation amputees) (holotype 209, P1 219, P2 220, P3 191, P5 260). First dorsal pore in 5/6. Prostomium epilobous. Setae closely paired. Setal papillae were not recognized (in holotype, P1, P2, P3) or setal papillae *ab* on segments 8–12 or 8–13 (P5). Clitellum on 24,25,26–36,37,38 (25–37 holotype and P1, 24–37 P2, 26–37 P3, 26–37 P4, 26–38 P5), involving twelve to fourteen segments. Tubercula pubertatis on 29,30–36 (holotype, P1, P2, P3 30–36, P5 ½29–36). Spermatheca pores paired in 9/10 & 10/11 in *cd*. Female pores on 14 above setae *b*. Male pores on 15 between setae *b* and *c*, confined to the segment and with (P5) or without (holotype, P1, P2, P3) small tumescences. Setal ratio at segment 12, in adult specimens *aa*: 6.4; *ab*: 1; *bc*: 4; *cd*: 0.8; *dd*: 20.8; *U*: 29.8 after clitellum *aa*: *ab*: *bc*: *cd*: *dd*: *U* = 5.5: 1: 2.2: 0.7: 18.8: 25 (S3 Table and Table 2).

**Table 2:** Setal ratio of *Eophila crodabepis* sp. nov. specimens and its mean on 12th segment.

SAMPLE	*aa*	*ab*	*bc*	*cd*	*dd*	*U*
***Crevada 1***	5.2	1	3.2	0.7	21.0	26.0
***Crevada 2***	5.0	1	4.2	0.8	20.0	23.0
***Crevada 3***	5.5	1	2.2	0.7	18.8	25.0
***Crevada 4***	6.0	1	6.0	0.8	20.0	22.0
***Crevada 5***	5.2	1	3.5	0.8	20.0	32.0
***Fratte 50***	6.0	1	3.5	0.8	19.0	28.0
***Grappa Mount 2***	6.8	1	4.2	0.6	20.0	38.4
***Praderadego 24***	7.0	1	4.0	0.8	21.0	28.0
***Praderadego 25***	6.0	1	3.5	0.7	21.0	31.0
***Roncavezzai 11***	7.0	1	5.0	0.8	27.0	28.0
***Roncavezzai 18***	4.3	1	3.2	0.8	19.5	23.0
***Roncavezzai 2***	7.0	1	4.0	0.9	19.0	25.0
***Roncavezzai 5***	6.0	1	3.7	0.8	25.0	27.0
***Val Posan 1***	7.0	1	4.0	0.9	23.0	27.0
***Val Posan 2***	6.8	1	4.7	0.5	18.7	37.9
***Val Posan 3***	7.0	1	3.0	0.5	18.0	37.0
***Val Posan 4***	8.0	1	5.0	0.9	23.0	38.0
***Val Posan 5***	7.0	1	4.0	0.8	19.5	30.0
***Ekar 5***	8.0	1	4.5	0.7	22.5	40.0
***HNHM 12678***	8.1	1	4.5	0.6	20.0	40.3
**MEAN**	6.4	1	4.0	0.7	20.8	30.3

Septa 5/6–7/8, 12/13–14/15 thickened, 8/9–11/12 strongly strengthened. Excretory system holoic, first pair of nephridia in 4. Nephridial bladders from 6 proclinate J-shaped; after the clitellum almost U-shaped. Hearts 6–11 with a pair of extraoesophageal vessels in 12. Calciferous glands in 10–12 with large vertical diverticula in 10. Crop large in 15–16 and muscular gizzard in 17–19. Typhlosole large, trifid. Testes and male funnels in 10 & 11 seemingly free. Vesicles four pairs in 9–12, the first two pairs are quite small, easy to overlook, those in 11 & 12 large. Spermathecae globular in 10 & 11. Ovaries moderate, pear-shaped in 13, ovarial sac moderate in 14, pendant from septum 13/14. Typhlosole trilobed in Crevada specimen (*Crevada 6*) commencing in 45 and terminating nine segment from pygidium (S3 Table) or begins around segment 23 and ends in segment 204 in the *Grappa Mount 2* specimen. Cross section of longitudinal musculature pinnate (S3 Fig).

*Eophila crodabepis* sp. nov. specimens collected in Treviso and Vicenza provinces were found alone, or in association with other species (S1 Table). It was collected in association with deep-burrowing species *Octodrilus complanatus* and *O*. *pseudocomplanatus* and with species belonging to other ecological categories, viz, *Aporrectodea sineporis* (Omodeo, 1952), *Octodrilus lissaensis*, *Eisenia spelaea* (Rosa, 1901) [12], *Aporrectodea caliginosa* and *Lumbricus rubellus*.

Analysing the sequences produced for this paper along with those of the other species of Lumbricidae in the previous publication Porco et al. 2013 [40], we were able to recover three MOTUs corresponding to *P*. *gestroi*, *E*. *tellinii* and the new species *E*. *crodabepis* sp. nov. using a 11% threshold value (data not shown). The mean intraspecific divergence found in these three species (*P*. *gestroi* 4.79% (ranging from 0% to 8.19%), *E*. *tellinii* 1.25% (ranging from 0% to 4.09%), *E*. *crodabepis* sp. nov. 4.55% (ranging from 0% to 7.43%), contrasted with a high interspecific mean divergence reaching 18.64% (range 13.86% to 21.97%—Fig 1) confirming the existence of a clear barcode gap for the dataset (Table 3). These ranges of genetic divergence are consistent or exceed those measured among species in Lumbricidae in previous studies [40,41] further confirming the separate specific status of the two taxa concerned.

**Table 3:** Intra-interspecific divergence between *Eophila crodabepis* sp. nov. and *Eophila tellinii* using *Perelia gestroi* as outgroup (note threshold value of 13.86%).

Species	Intraspecific	Interspecific		
		*E*. *crodabepis*	*E*. *tellinii*	*P*. *gestroi*
***Eophila crodabepis* sp. nov.**	4.55			
***Eophila tellinii***	1.25	13.86		
***Perelia gestroi***	4.79	20.10	21.97	

### Comments

**Fig 5:**
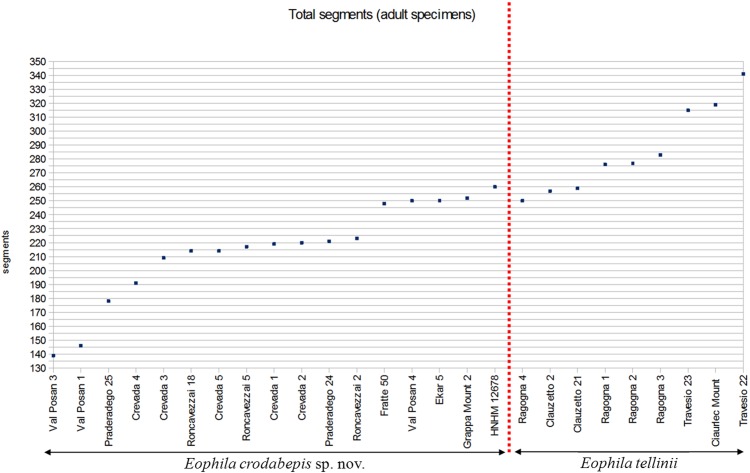
Adult specimens total segment counts. Data of the adult specimens which were probably damaged by predation (*Roncavezzai 11*, *Val Posan 2*, *Val Posan 5*) were not included.

**Fig 6:**
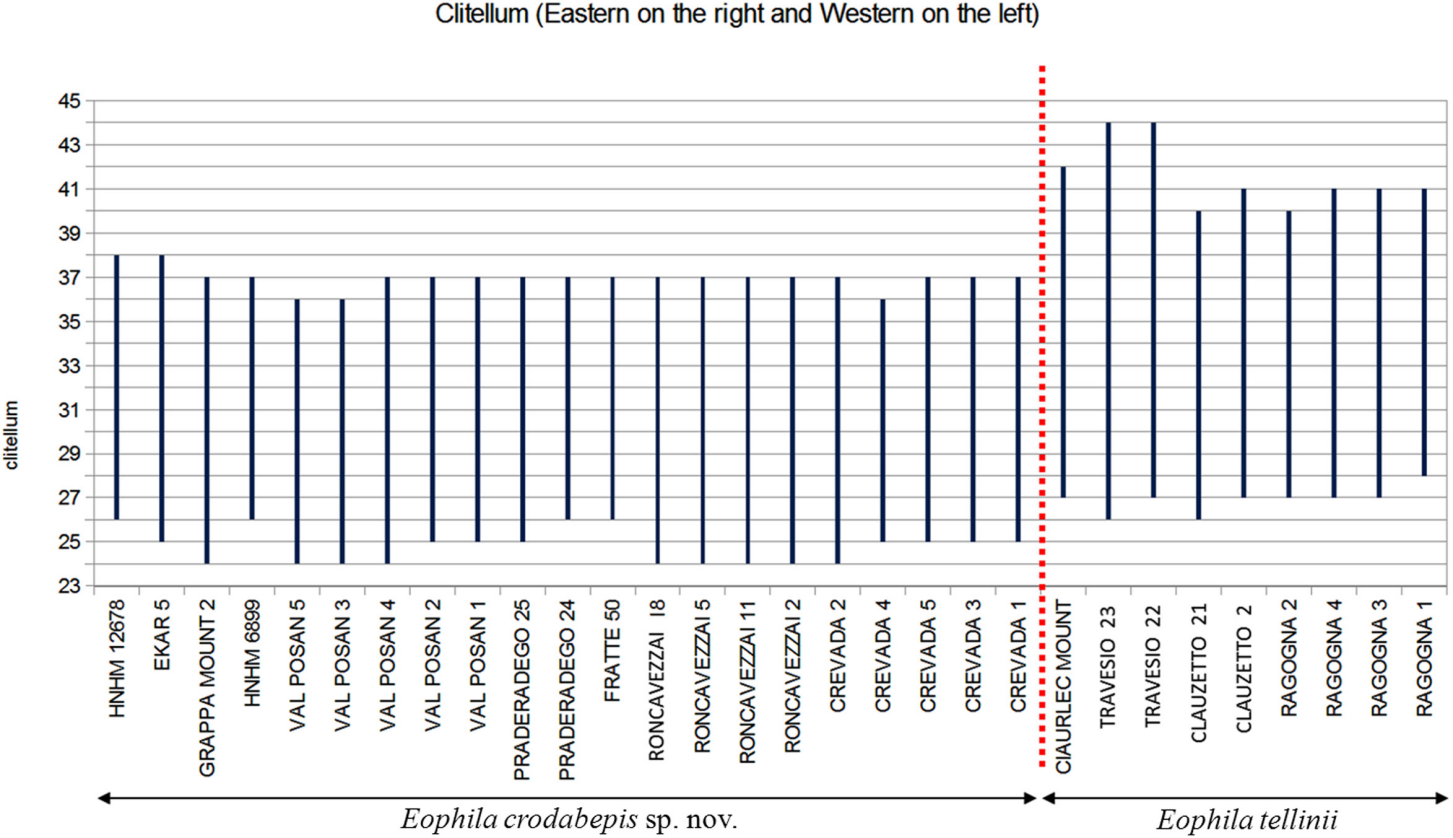
Clitellum location of adult *Eophila tellinii* and *Eophila crodabepis* sp. nov. specimens with geographic distribution from East to West.

The new species differs from *E*. *tellinii* in its smaller mean size (100–230 vs. 170–360 mm), lower number of segments (139–252 vs. 250–341) (Fig 5), lesser weight (2.6–12.5 vs. 11.4–28.2 g) and different locations of clitellum and tubercula pubertatis. In *E*. *crodabepis* sp. nov. the clitellum covers segments 24,25,26–36,37,38 and tubercles of puberty are on segments 29,30–36 instead of 26,27–41 and 32–37 as in the syntype of *E*. *tellinii* (Rosa, 1888) (Fig 6). In other *E*. *tellinii* specimens, the clitella are located on segments 27–40, 27–41, 26–40, 27–42, 27–44 and 26–44 (S3 Table), which are within range of the position described by Rosa as on segments 27–41. The same applies to the tubercula pubertatis found on segments ½29,29,30–36 in *E*. *crodabepis* sp. nov., instead of 30,31,32–37,38,39,40 in *E*. *tellinii* (S3 Table). Also, the average weight, length and number of segments of *Eophila crodabepis* sp. nov. is lower compared with (*E*. *tellinii*) specimens collected in Friuli Venezia Giulia (S3 Table). Many other features appear the same between the two species, such as the coloration, the shape of the prostomium, the position of the first dorsal pore, and the body shape (S3 Table).

**Fig 7:**
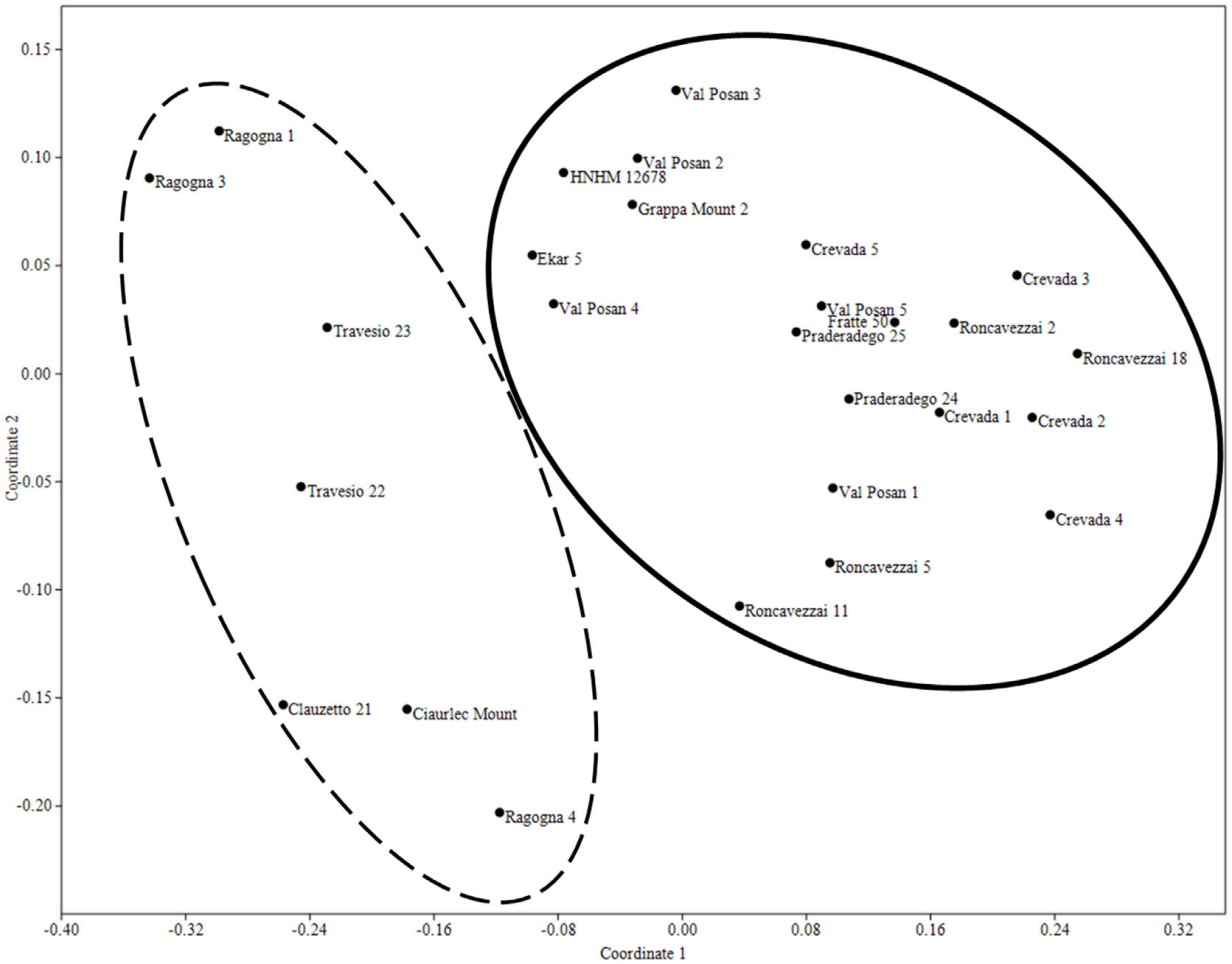
NMDS scatter plot of the Setal ratio in *E*. *tellini* and *E*. *crodabepis* sp. nov. specimens. Splitting between *E*. *tellinii* group (broken line) and *E*. *crodabepis* group (solid line) (Stress = 0.06956; First axis R^2^ = 0.8664). The two groups have different setal ratios.

NMDS analysis for the setal ratios of the of *E*. *tellinii* and *E*. *crodabepis* sp. nov. show the two groups are evidently different (Fig 7). T- test (Tables 1 and 2) evaluate the NMDS analysis and, except for *cd*, all setal ratios are significantly different for p<0.05 or p<0.001 (Table 4).

**Table 4:** T-test of the setal ratios of the two groups.

T test	aa	ab	bc	cd	dd	U
**p(value)**	*	n.s.	*	n.s.	***	**

Differences between two groups *E*. *tellinii* and *E*. *crodabepis* sp. nov. for P(value)<0.05 *. Setal ratio dd significant for P(value)<0.001***. cd ratio appears with no significant differences (ab is used for standard unit in each groups). P(value)<0.05 *; P(value) <0.01 **; P(value)<0.001***.

From these analyses it is possible to establish feature differences between *E*. *tellinii* (which is present in Friuli Venezia Giulia) and the other population living in different locations in Veneto. Previously, all the specimens were field identified as *E*. *tellinii* based on typical colouration however, clitella and tubercula pubertatis location as well as the biometry are now proven to be different.

## Discussion

### Ecological observations

- Some specimens have been victims of predation and one of the causes is the leech collected in Val Posan and Roncavezzai: *Haemopis sanguisuga* were found at the collection places.
- The fact that *Octodrilus complanatus* and *O*. *pseudocomplanatus* specimens were collected together with *Eophila tellinii* (S1 Table) is interesting as they are both classed as deep-burrowing species.
- The new species is defined on differences both in morpho-genetic characters as well as in its geographic range [42].
- Differences in DNA are used to separate the earthworm species based on their primary types and topotypes as initially advocated in Blakemore *et al*. [43] allowing full species characterization as in Blakemore [44].

## Conclusions

*Eophila crodabepis* sp. nov. is clearly distinguished from *Eophila tellinii* both for diagnostic morphological characters (Figs 5 and 7) and for the definitive genetic data (Fig 1) plus their geographical distributions (Figs 2 and 6). Its superficial affinity with *Eophila tellinii*, especially in macro-morphology (bands brown-purple), led to initial misidentification in the field as *Eophila tellinii* [5,12,13]. Further ecological assessment is now possible on the objectively differentiated taxa.

## Supporting Information

S1 Fig *Eophila tellinii* morphological and anatomical details. Livery pattern in Travesio specimen and peristomial detail (*Ragogna 1* specimen). 7.98 μm and 4.35 μm ventral, dorsal and lateral views of specimen (*Ragogna 2*); virtual sections of hindmost segments: Schematic and transversal sections of last ten segments lacking typhlosole, hindmost body segment- (middle) mesial-middle section (upper right), and sagittal medial section (bottom) from Clauzetto specimen.
(DOC)

S2 Fig *Eophila tellinii* ecological details. Wood near Travesio (PN) in which *Eophila tellini* was found along with its casts.
(DOC)

S3 Fig *Eophila crodabepis* sp. nov. anatomical details. 7.98 μm on ventral, dorsal and lateral views and 3.08μm from Crevada (*Crevada 6*). DataViewer´s virtual sections of hindmost segments: Schematic and transversal sections of last ten segments where no typhlosole occurs; hindmost body segment- (middle) mesial-middle section (upper right), and sagittal medial section (bottom). In the middle right is a transversal sections at level of the penultimate segment; plus cross section of longitudinal pinnate musculature (*HNHM 6899* specimen).
(DOC)

S1 Table Geographical and ecological information for each specimen analyzed. (DOC)

S2 Table Morphological and anatomical features from the literature. (DOC)

S3 Table Morphology and anatomy of specimens inspected and from the literature. (DOC)
