# Correction: Barcoding *Eophila crodabepis* sp. nov. (Annelida, Oligochaeta, Lumbricidae), a Large Stripy Earthworm from Alpine Foothills of Northeastern Italy Similar to *Eophila tellinii* (Rosa, 1888)

**DOI:** 10.1371/journal.pone.0160218

**Published:** 2016-08-01

**Authors:** Maurizio G. Paoletti, Robert J. Blakemore, Csaba Csuzdi, Luca Dorigo, Angelo Leandro Dreon, Federico Gavinelli, Francesca Lazzarini, Nicola Manno, Enzo Moretto, David Porco, Enrico Ruzzier, Vladimiro Toniello, Andrea Squartini, Giuseppe Concheri, Marina Zanardo, Javer Alba-Tercedor

There are errors in several figure captions in the published article. The name of the species *E*. *tellinii* is spelled incorrectly in the captions for Fig 7 and S2 Fig. The captions for S1 and S3 Figs are missing their component designations. Please view correct captions for Fig 7 and S2 Fig here. The correctly labelled S1 and S3 Figs and their captions can also be found here.

**Fig 7. NMDS scatter plot of the Setal ratio in *E*. *tellinii* and *E*. *crodabepis* sp. nov. specimens.** Splitting between *E*. *tellinii* group (broken line) and *E*. *crodabepis* group (solid line) (Stress = 0.06956; First axis R^2^ = 0.8664). The two groups have different setal ratios.

In references 12 and 15 the species name was not properly italicized. The correct references are:

12. Braido V, Ecologia e distribuzione geografica di un lombricide di grandi dimensioni: *Eophila tellinii* (Rosa, 1888), Tesi di laurea discussa alla Facoltà di Scienze Biologiche, Università degli Studi di Padova,1993.

15. Braido V, Toniello V, Paoletti MG, *Eophila tellinii* (Rosa, 1888): inconsueto lombrico variopinto di grandi dimensioni. Speleologia Veneta, 1997; (5): 122–130.

## Supporting Information

S1 Fig *Eophila tellinii* morphological and anatomical details. a)Livery pattern in Travesio specimen and b) peristomial detail (*Ragogna 1* specimen); c)7.98 µm and 4.35 µm ventral, dorsal and lateral views of specimen (*Ragogna 2*); d)virtual sections of hindmost segments: schematic and transversal sections of last ten segments lacking typhlosole, hindmost body segment- (middle) mesial-middle section (upper right), and sagittal medial section (bottom) from Clauzetto specimen.
(DOC)

S2 Fig *Eophila tellinii* ecological details. Wood near Travesio (PN) in which *Eophila tellinii* was found along with its casts.
(DOC)

S3 Fig *Eophila crodabepis* sp. nov. anatomical details. a)7.98 µm on ventral, dorsal and lateral views and 3.08µm from Crevada (*Crevada 6*); b)virtual sections of hindmost segments: schematic and transversal sections of last ten segments where no typhlosole occurs; hindmost body segment- (middle) mesial-middle section (upper right), and sagittal medial section (bottom); transversal sections at level of the penultimate segment (middle right); c)cross section of longitudinal pinnate musculature (*HNHM 6899* specimen).
(DOC)
