# Supplementary figures and images for: Barcoding Eophila crodabepis sp. nov. (Annelida, Oligochaeta, Lumbricidae), a Large Stripy Earthworm from Alpine Foothills of Northeastern Italy Similar to Eophila tellinii (Rosa, 1888)
Source: PLoS One. 2016 Mar 28;11(3):e0151799. doi: 10.1371/journal.pone.0151799 (PMC4809493; doi:10.1371/journal.pone.0151799)

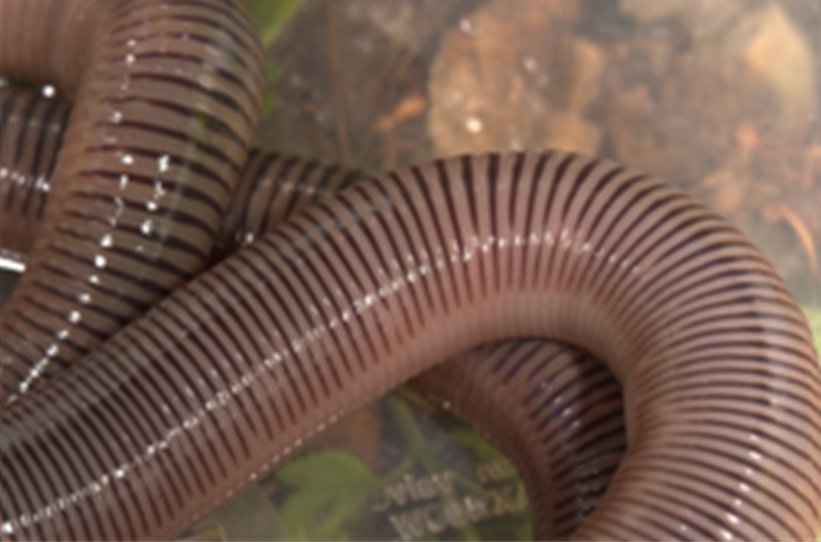


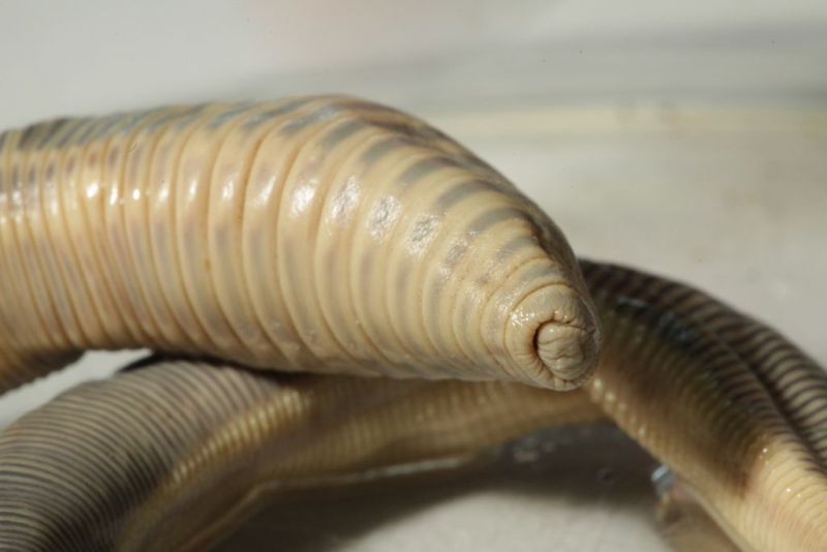


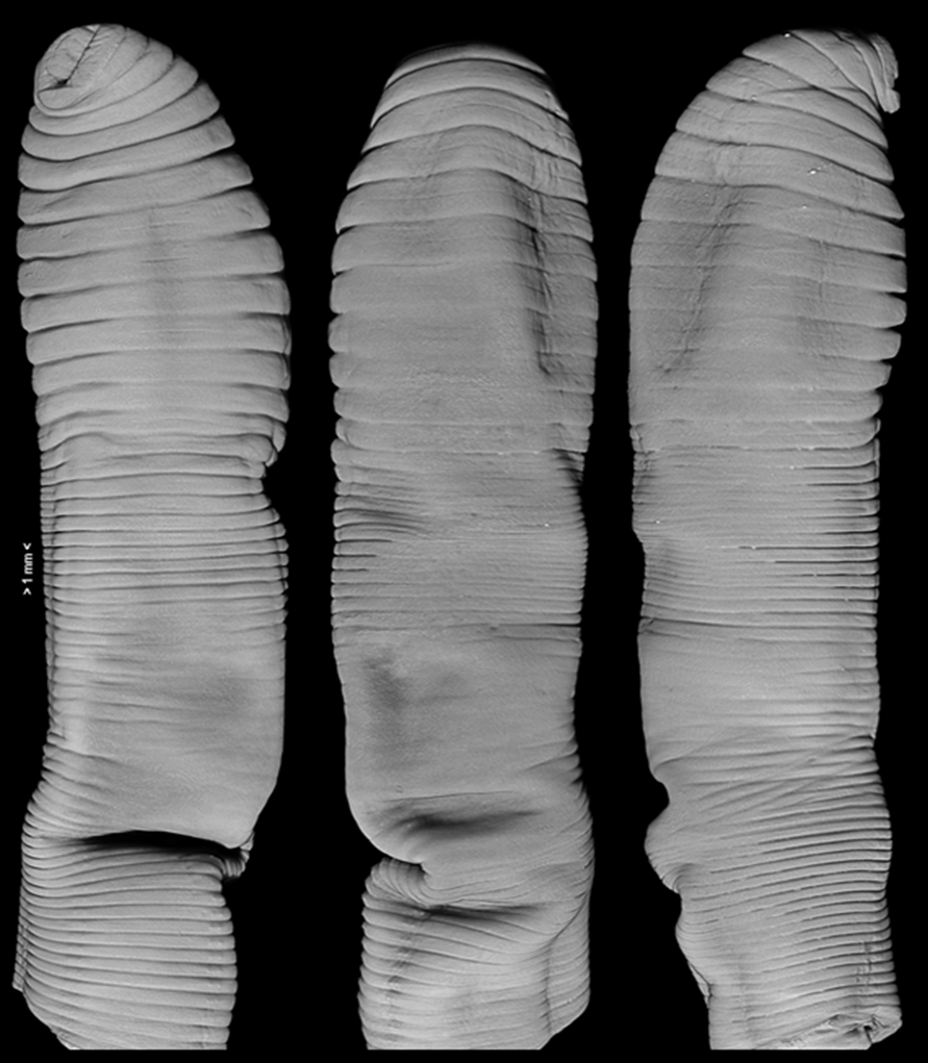


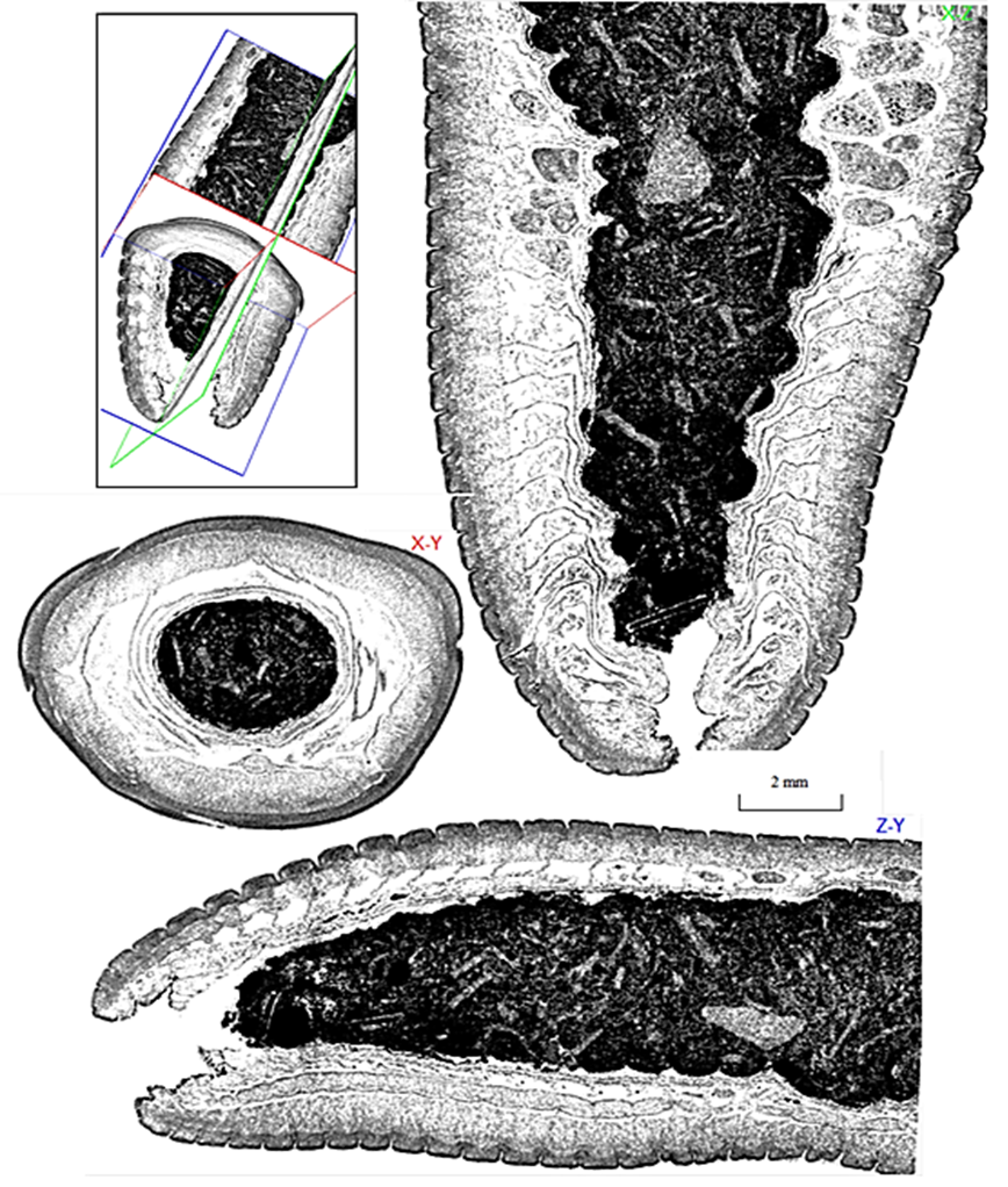

Supplement: S1 Fig — Livery pattern in Travesio specimen and peristomial detail (Ragogna 1 specimen). 7.98 μm and 4.35 μm ventral, dorsal and lateral views of specimen (Ragogna 2); virtual sections of hindmost segments: Schematic and transversal sections of last ten segments lacking typhlosole, hindmost body segment- (middle) mesial-middle section (upper right), and sagittal medial section (bottom) from Clauzetto specimen. (DOC) [file pone.0151799.s001.doc]

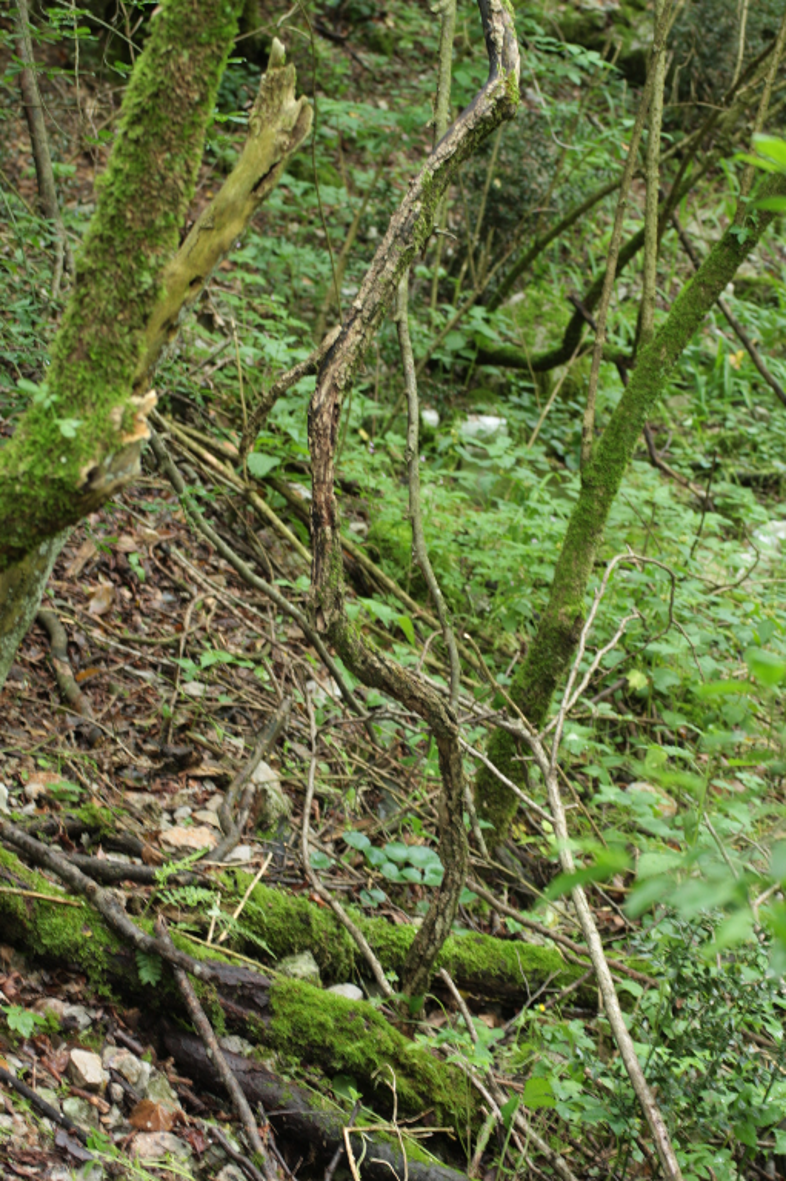


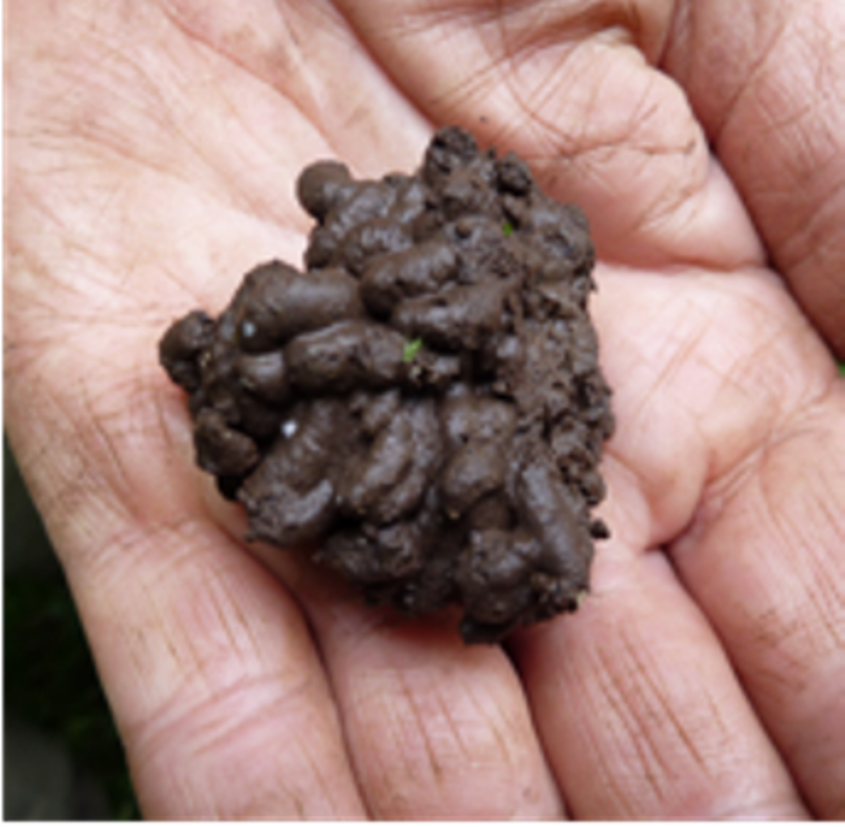

Supplement: S2 Fig — Wood near Travesio (PN) in which Eophila tellini was found along with its casts. (DOC) [file pone.0151799.s002.doc]

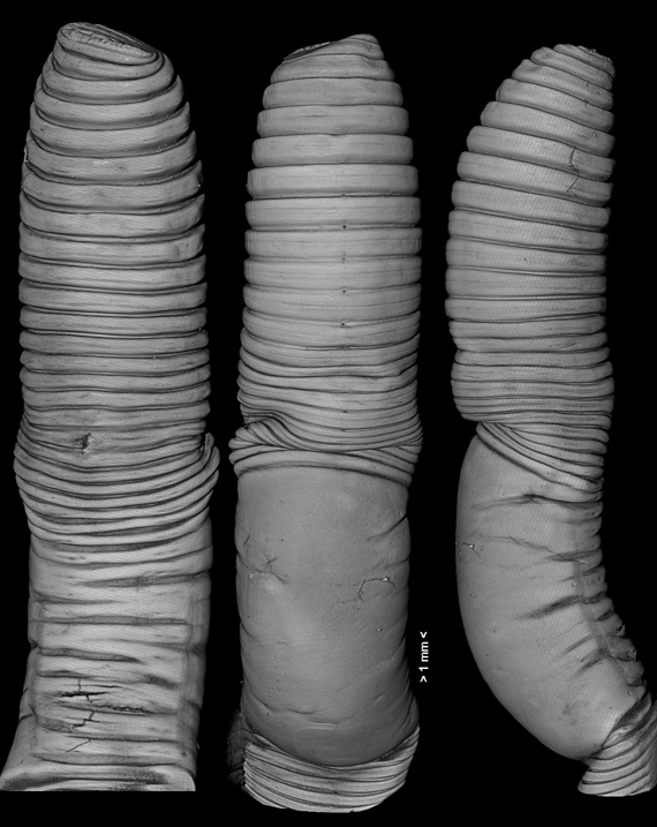


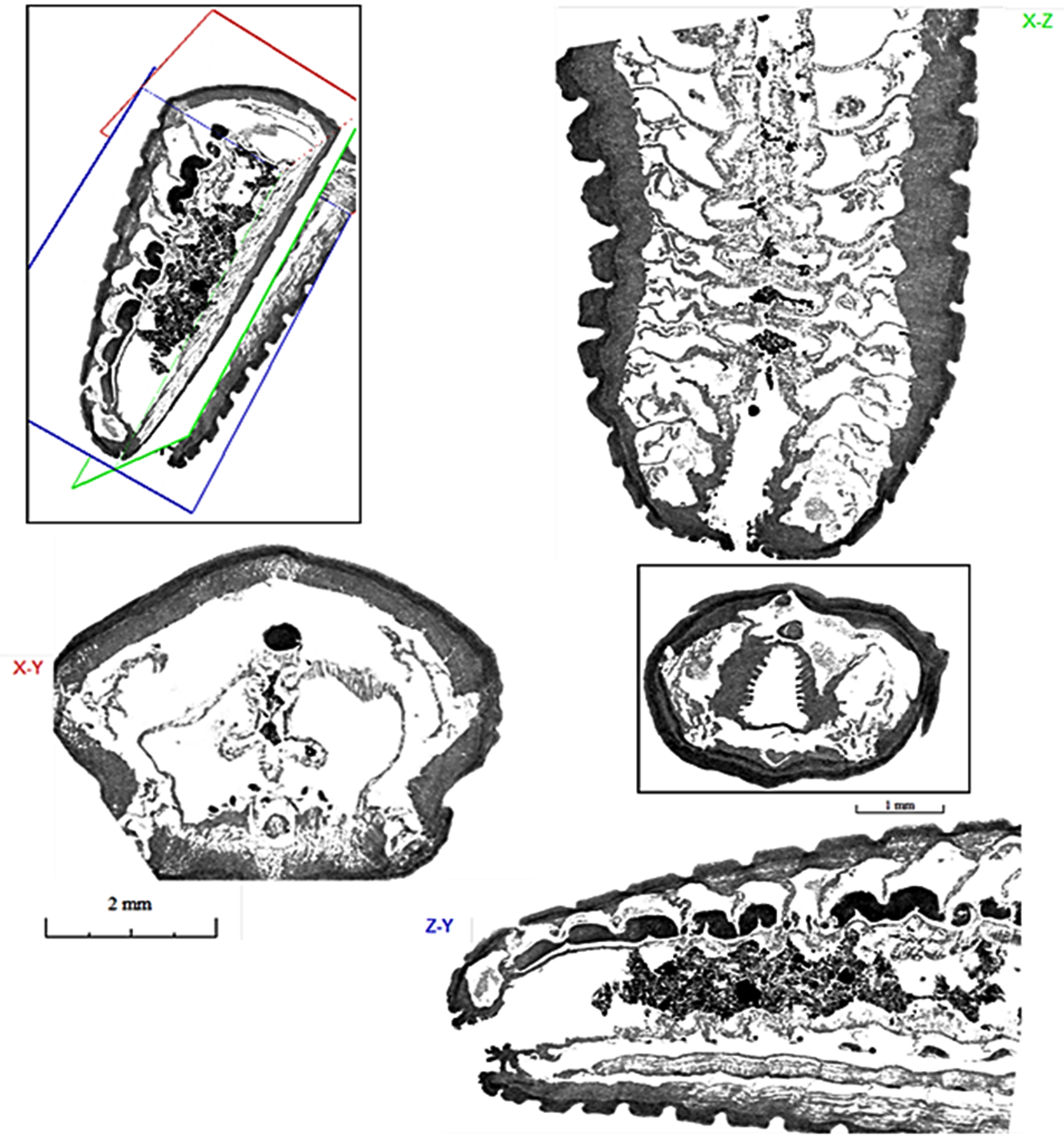


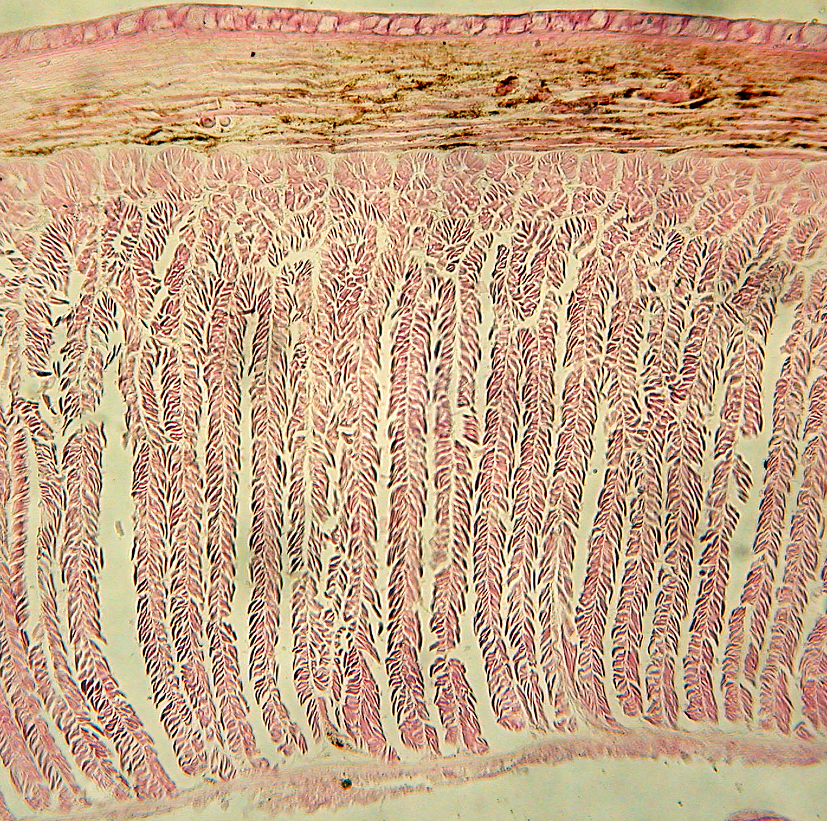

Supplement: S3 Fig — 7.98 μm on ventral, dorsal and lateral views and 3.08μm from Crevada (Crevada 6). DataViewer´s virtual sections of hindmost segments: Schematic and transversal sections of last ten segments where no typhlosole occurs; hindmost body segment- (middle) mesial-middle section (upper right), and sagittal medial section (bottom). In the middle right is a transversal sections at level of the penultimate segment; plus cross section of longitudinal pinnate musculature (HNHM 6899 specimen). (DOC) [file pone.0151799.s003.doc]
