# Supplementary material for: Barcoding Eophila crodabepis sp. nov. (Annelida, Oligochaeta, Lumbricidae), a Large Stripy Earthworm from Alpine Foothills of Northeastern Italy Similar to Eophila tellinii (Rosa, 1888)
Source: PLoS One. 2016 Mar 28;11(3):e0151799. doi: 10.1371/journal.pone.0151799 (PMC4809493; doi:10.1371/journal.pone.0151799)
Supplement: S1 Table — (DOC) [file pone.0151799.s004.doc]

**S1 Table**. Geographical and ecological information datas of each specimens analysed.

| **SPECIMENS CODE** | **COLLECTING SITE** | **SECTOR** | **PROVINCE** | **REGION** | **COUNTRY** | **LATITUDE** | **LONGITUDE** | **ALTITUDE (m a.s.l.)** | **COLLECTION DATE** | **HABITAT** | **DOMINANT PLANT SPECIES** | **LITHOLOGY** | ***Eophila tellinii* TOTAL specimens** | ***Eophila tellinii* ADULT specimens** | ***Eophila tellinii* IMMATURE specimens** | ***Eophila tellinii* SUBADULT specimens** | ***Eophila tellinii (*specimens/m²)** | ***Octodrilus complanatus* (specimens/m²)** | ***Octodrilus pseudocomplanatus (specimens/m²)*** | ***Octodrilus lissaensis* (specimens/m²)** | ***Octodrilus phaenoemiandrum* (specimens/m²)** | ***Octodrilus sp.* (specimens/m²)** | ***Octolasium lacteum* (specimens/m²)** | ***Octolasium sp.* (specimens/m²)** | ***Aporrectodea sp.* (specimens/m²)** | ***Eiseniona sineporis* (specimens/m²)** | ***Eisenia spelaea* (specimens/m²)** | ***immature specimens* (specimens/m²)** | **COLLECTOR** | **REFERENCES** |
| --- | --- | --- | --- | --- | --- | --- | --- | --- | --- | --- | --- | --- | --- | --- | --- | --- | --- | --- | --- | --- | --- | --- | --- | --- | --- | --- | --- | --- | --- | --- |
| **Ragogna 1,2,3,4,5** | Ragogna Hills | Ragogna | UD | Friuli Venezia Giulia | Italy | 46°11'6.80"N | 12°58'34.12"E | 350 | 12/11/2013 | N.D. | N.D. | N.D. | 5 | 4 | 1 | 0 | 2,5 | / | / | / | / | / | / | / | / | / | / | / | Maurizio G. Paoletti | / |
| **Altopiano d' Asiago 1** | Altopiano d'Asiago | Asiago | VI | Veneto | Italy | N.D | N.D | 750 | 02/07/1980 | hazel, maple and chestnut wood | N.D. | stony soil | 1 | 1 | 0 | 0 | N.D. | N.D. | N.D. | N.D. | N.D. | N.D. | N.D. | N.D. | N.D. | N.D. | N.D. | N.D. | Bonifazi | Omodeo, 1988 |
| **Altopiano d' Asiago 2** | Altopiano d'Asiago | Asiago | VI | Veneto | Italy | N.D | N.D | 650 | 12/07/1980 | some habitat | N.D. | N.D. | 1 | 0 | 1 | 0 | N.D. | N.D. | N.D. | N.D. | N.D. | N.D. | N.D. | N.D. | N.D. | N.D. | N.D. | N.D. | Omodeo & Valbusa Dall'Armi | Omodeo, 1988 |
| **Clauzetto 21** | Celante | Clauzetto | PN | Friuli Venezia Giulia | Italy | 46°14'32"N | 12°55'25"E | 410 | 10/10/2010 | wood | N.D. | N.D. | 1 | 1 | 0 | 0 | 0,5 | 0,5 | / | / | / | 2,5 | / | / | 0,5 | / | / | / | Leandro Dreon | / |
| **Clauzetto 2** | Pradis di Sotto | Clauzetto | PN | Friuli Venezia Giulia | Italy | 46°14'3.304"N | 12°54'3.748"E | 584 | 18/03/2015 | N.D. | N.D. | N.D. | 1 | 1 | 0 | 0 | 0,5 | / | / | / | / | / | / | / | / | / | / | / | Luca Dorigo | / |
| **Travesio 22,23** | Borgata Praforte | Travesio | PN | Friuli Venezia Giulia | Italy | 46°12'24"N | 12°52'40"E | 224 | 25/11/2010 | N.D. | N.D. | N.D. | 2 | 2 | 0 | 0 | 1 | / | / | / | / | / | / | / | / | / | / | / | Leandro Dreon | / |
| **Ciaurlec Mount 1,2,3,4** | Ciaurlec Mount | Castelnuovo del Friuli | PN | Friuli Venezia Giulia | Italy | 46°21'20"N | 12°51'8.40"E | 1000 | 10/07/1994 | wood | Fagus sylvatica L., Fraxinus excelsior L., Populus tremula L., Ostrya carpinifolia L. | soil rich in limestone rock outcrops | 4 | 2 | 2 | 0 | 1,4 | / | / | / | / | 0,3 | / | / | / | / | / | / | Valentina Braido | Braido, 1993 |
| **Ciaurlec Mount** | Ciaurlec Mount | Ciaurlec Mount | PN | Friuli Venezia Giulia | Italy | 46°14'19.820"N | 12°48'58.133"E | 900 | 16/05/2010 | calcicole mesothermophilic beechwoods | N.D. | N.D. | 1 | 1 | 0 | 0 | 0,5 | / | / | / | 0,5 | 0,5 | 1 | / | / | / | / | / | Leandro Dreon | / |
| **Fornace Toppo 1,2** | Fornace Toppo | Travesio | PN | Friuli Venezia Giulia | Italy | 46°12'32.87"N | 12°49'28.36"E | 75 | 12/10/2014 | N.D. | N.D. | N.D. | 2 | 0 | 2 | 0 | 1 | / | / | / | / | / | / | / | / | / | / | / | Maurizio G. Paoletti and Leandro Dreon | / |
| **Toppo Carnico 1,2,3,4,5,6,7,8,9,10,11,12,13,14,15,16,17,18,19,20,21,22,23** | Toppo Carnico | Castelnuovo del Friuli | PN | Friuli Venezia Giulia | Italy | 46°12'29" N | 12°49'21.40" E | 285 | 17/10/1993 | wood | Quercus petraea Lieblein, Quercus pubescens W. , Quercus robur L., Prunus avium L., Fraxinus excelsior L., Acer pseudoplatanus L., and in the undergrowth by Sambucus nigra L., Corylus avellana L., Cornus sanguinea L. | reddish soil rich in clay and small limestone debris | 7 | 3 | 4 | 0 | 2,3 | / | 0,3 | / | / | 2,3 | / | / | / | / | / | / | Valentina Braido | Braido, 1993 |
|  | Toppo Carnico | Castelnuovo del Friuli | PN | Friuli Venezia Giulia | Italy | 46°12'29" N | 12°49'21.40" E | 285 | 19/04/1994 | wood | Quercus petraea Lieblein, Quercus pubescens W. , Quercus robur L., Prunus avium L., Fraxinus excelsior L., Acer pseudoplatanus L., and in the undergrowth by Sambucus nigra L., Corylus avellana L., Cornus sanguinea L. | reddish soil rich in clay and small limestone debris | 16 | 3 | 13 | 0 | 5,3 | / | 1 | 2 | / | 0,7 | / | / | / | / | / | / | Valentina Braido | Braido, 1993 |
| **Borgo Cilia 1,2,3,4,5,6,7,8,9,10,11** | Borgo Cilia | Meduno | PN | Friuli Venezia Giulia | Italy | 46°13'34" N | 12°49'6.40" E | 580 | 17/10/1993 | wood | Carpinus betulus L., Prunus avium L., Castanea sativa Miller, Acer campestre L., Alnus incana L., Laburnum anagyroides M., Ostrya carpinifolia S., Corylus avellana L. | soil full of very altered rocky debris | 8 | 6 | 2 | 0 | 2,7 | / | / | 2,3 | / | 2 | / | / | / | / | / | / | Valentina Braido | Braido, 1993 |
|  | Borgo Cilia | Meduno | PN | Friuli Venezia Giulia | Italy | 46°13'34" N | 12°49'6.40" E | 580 | 08/05/1994 | wood | Carpinus betulus L., Prunus avium L., Castanea sativa Miller, Acer campestre L., Alnus incana L., Laburnum anagyroides M., Ostrya carpinifolia S., Corylus avellana L. | soil full of very altered rocky debris | 3 | 1 | 2 | 0 | 1 | / | / | 1,3 | / | 2,7 | / | / | / | / | / | / | Valentina Braido | Braido, 1993 |
| **Crevada 1,2,3,4,5,6** | Crevada | Susegana | TV | Veneto | Italy | 45°52'18.89"N | 12°14'46.87"E | 80 | 16/11/2013 | N.D. | N.D. | N.D. | 6 | 6 | 0 | 0 | 3 | / | / |  | / |  | / | / | / | / | / | / | Maurizio G. Paoletti and Vladimiro Toniello | / |
| **Val Posan 1.0,2.0,3.0,4.0,5.0** | Val Posan | Montello | TV | Veneto | Italy | 45°49'52"N | 12°12'24.60"E | 140 | 22/10/1993 | sink-hole | Robinia pseudoacacia L. |  | 1 | 1 | 0 | 0 | 0,3 | / | / |  | / |  | / | / | / | / | / | / | Valentina Braido | Braido, 1993 |
|  | Val Posan | Montello | TV | Veneto | Italy | 46°49'52"N | 12°12'24.60"E | 140 | 23/04/1994 | sink-hole | Robinia pseudoacacia L. |  | 4 | 0 | 4 | 0 | 1,3 | / | 2 | 4,7 | / | 4 | / | / | / | / | / | / | Valentina Braido | Braido, 1993 |
| **Val Posan 1,2,3,4,5,6,7,8,9** | Val Posan | Crocetta del Montello | TV | Veneto | Italy | 45°50'30.50"N | 12°04'02.40"E | 146 | 31/05/2012 | sink-hole | N.D. | N.D. | 9 | 5 | 4 | 0 | 1,8 | / | / | / | / | / | / | / | / | / | / | 1,2 | Maurizio G. Paoletti | / |
| **Villa Jacur 1,2,3** | Villa Jacur | Susegana | TV | Veneto | Italy | 45°51'27.57"N | 12°12'43.12"E | 77 | apr-13 | N.D. | N.D. | N.D. | 3 | 0 | 3 | 0 | 1,5 | / | / | / | / | / | / | / | / | / | / | / | Maurizio G. Paoletti and Vladimiro Toniello | / |
| **S. Boldo Pass** | S. Boldo Pass |  | TV | Veneto | Italy | N.D | N.D | N.D | 16/05/1973 | chestnut wood | N.D. | stony soil | 1 | 0 | 0 | 1 | N.D. | / | / | / | / | / | / | / | / | / | / | / | Alessandro Minelli | Omodeo, 1988 |
| **Tavaran Grande 1,2,3,4,5,6,7** | Tavaran Grande | Montello | TV | Veneto | Italy | 46°50'40"N | 12°9'3.60"E | 100 | 30/04/1994 | karstic cavity | Robinia pseudoacacia L. | red soil | 4 | 0 | 4 | 0 | 1,3 | 0,3 | 0,7 | 3 | / | / | / | / | / | 0,7 | / | / | Valentina Braido | Braido, 1993 |
|  | Tavaran Grande | Montello | TV | Veneto | Italy | 46°50'40"N | 12°9'3.60"E | 100 | 12/10/1994 | karstic cavity | Robinia pseudoacacia L. | red soil | 3 | 1 | 2 | 0 | 1 | 0,7 | 0,7 | 3 | / | 3,3 | / | 0,3 | / | / | / | / | Valentina Braido | Braido, 1993 |
| **Follina**  **1, 2, 3, 4, 5, 6, 7** | Follina | Cison di Valmarino | TV | Veneto | Italy | 46°56'27"N | 12°8'46.6"E | 230 | 16/10/1993 | wood | Robinia pseudoacacia L., Corylus avellana L. and Carpinus betulus L. | heavy clay grey soil | 2 | 2 | 0 | 0 | 0,7 | / | 0,7 | 0,3 | / | 0,7 | / | / | / | / | / | / | Valentina Braido | Braido, 1993 |
|  | Follina | Cison di Valmarino | TV | Veneto | Italy | 46°56'27"N | 12°8'46.6"E | 230 | 25/04/1994 | wood | Robinia pseudoacacia L., Corylus avellana L. and Carpinus betulus L. | heavy clay grey soil | 5 | 4 | 1 | 0 | 1,6 | / | 1,3 | 1 | / | 0,3 | / | / | / | / | 0,3 | / | Valentina Braido | Braido, 1993 |
| **Roncavezzai 7** | Roncavezzai | Follina | TV | Veneto | Italy | 45°57'10.75"N | 12°7'5.91"E | 187 | May 2012 | N.D. | N.D. | N.D. | 1 | 0 | 1 | 0 | 0,5 | / | / | / | / | / | / | / | / | / | / | / | Maurizio G. Paoletti | / |
| **Roncavezzai 1,2,18** | Roncavezzai | Follina | TV | Veneto | Italy | 45°57'10.75"N | 12°7'5.91"E | 187 | October 2012 | N.D. | N.D. | N.D. | 3 | 2 | 1 | 0 | 1,5 | / | / | / | / | / | / | / | / | / | / | / | Maurizio G. Paoletti | / |
| **Roncavezzai 6,10** | Roncavezzai | Follina | TV | Veneto | Italy | 45°57'10.75"N | 12°7'5.91"E | 187 | 27/05/2012 | N.D. | N.D. | N.D. | 2 | 0 | 2 | 0 | 1 | / | / | / | / | / | / | / | / | / | / | 9 | Maurizio G. Paoletti | / |
| **Roncavezzai 11** | Roncavezzai | Follina | TV | Veneto | Italy | 45°57'10.75"N | 12°7'5.91"E | 187 | 07/11/2010 | N.D. | N.D. | N.D. | 1 | 1 | 0 | 0 | 0,5 | / | / | / | / | / | / | / | / | / | / | / | Maurizio G. Paoletti | / |
| **Roncavezzai 9** | Roncavezzai | Follina | TV | Veneto | Italy | 45°57'10.75"N | 12°7'5.91"E | 187 | 01/11/2010 | N.D. | N.D. | N.D. | 1 | 0 | 1 | 0 | 0,5 | / | / | / | / | / | / | / | / | / | / | / | Maurizio G. Paoletti | / |
| **Roncavezzai 3,5,8** | Roncavezzai | Follina | TV | Veneto | Italy | 45°57'10.75"N | 12°7'5.91"E | 187 | 20/10/2012 | N.D. | N.D. | N.D. | 3 | 1 | 2 | 0 | 1,5 | / | / | / | / | / | / | / | / | / | / | / | Maurizio G. Paoletti | / |
| **Fratte 50** | Fratte | Follina | TV | Veneto | Italy | 45°59'31.71"N | 12°20'9.72"E | 62 | 21/04/1987 | N.D. | N.D. | N.D. | 1 | 1 | 0 | 0 | 0,5 | / | / | / | / | / | / | / | / | / | / | / | Maurizio G. Paoletti | / |
| **Praderadego 24,25** | Praderadego | Val Mareno | TV | Veneto | Italy | 45°58'45"N | 12°7'32"E | 400 | 20/11/2011 | N.D. | N.D. | N.D. | 2 | 2 | 0 | 0 | 2 | / | / | / | / | 1 | / | / | / | / | / | / | Maurizio G. Paoletti | / |
| **Cesen 6** | Cesen Mount | Pianezze di Valdobbiadene | TV | Veneto | Italy | 45°55'49.62"N | 12° 0'27.77"E | 890 | 14/09/2014 | deciduous wood | Corylus avellana L. and Acer campestre L. | N.D. | 1 | 0 | 1 | 0 | 0,5 | / | / | / | / | / | / | / | / | / | / | / | Maurizio G. Paoletti | / |
| **Valdobbiadene 1,2** | Valdobbiadene | Valdobbiadene | TV | Veneto | Italy | N.D | N.D | 450 | 08/04/1972 | chestnut wood | N.D. | N.D. | 2 | 0 | 1 | 1 | N.D. | N.D. | N.D. | N.D. | N.D. | N.D. | N.D. | N.D. | N.D. | N.D. | N.D. | N.D. | Alessandro Minelli | Omodeo, 1988 |
| **Grappa Mount 1,2** | Grappa Mount | Grappa Mount | TV | Veneto | Italy | 45°51'52.10"N | 11°46'21.91"E | 1230 | 02/07/2011 | N.D. | N.D. | grey-brown soil | 2 | 1 | 1 | 0 | 1 | / | / | / | / | / | / | / | / | / | / | 1,5 | Maurizio G. Paoletti and Enrico Ruzzier | / |
| **Campo Solagna 17** | Campo Solagna | Grappa Mount | TV | Veneto | Italy | 45°50'22"N | 11°45'04"E | 1000 | 03/07/2011 | N.D. | N.D. | N.D. | 1 | N.D. | N.D. | N.D. | N.D. | / | / | / | / | / | / | / | / | / | / | / | Maurizio G. Paoletti and Enrico Ruzzier | / |
| **Ekar 5** | Ekar Mount | Asiago | VI | Veneto | Italy | 45°50'47.08"N | 11°34'6.24"E | 1300 | 10/11/2014 | N.D. | N.D. | N.D. | 1 | 1 | 0 | 0 | 0,5 | / | / | / | / | / | / | / | / | / | / | / | Maurizio G. Paoletti and Enzo Moretto | / |
| **Pradis 1** | Pradis | Clauzetto | PN | Friuli V. G. | Italy | 46°14'3.656"N | 12°54'12.56"E | N.D | 01/03/2015 | N.D. | N.D. | N.D. | 1 (observed in nature) | N.D. | N.D. | N.D. | / | / | / | / | / | / | / | / | / | / | / | / | Luca Dorigo | / |
| **Pradis 2** | Pradis | Clauzetto | PN | Friuli V. G. | Italy | 46°13'57.166"N | 12°54'27.169"E | N.D | 01/03/2015 | N.D. | N.D. | N.D. | 1 (observed in nature) | N.D. | N.D. | N.D. | / | / | / | / | / | / | / | / | / | / | / | / | Luca Dorigo | / |
| **Casasola** | Casasola | Frisanco | PN | Friuli V. G. | Italy | 46°13'9.628"N | 12°43'28.060"E | 380 | April 2013 | wood | Quercus petraea Lieblein | N.D. | 1 (observed in nature) | N.D. | N.D. | N.D. | / | / | / | / | / | / | / | / | / | / | / | / | Luca Dorigo | / |
| **HNHM 6899** | Mt. Cesen | Valdobbiadene | TV | Veneto | Italy | ND | ND | 1000 | 09/10/1969 | ND | ND | ND | ND | ND | ND | ND | ND | ND | ND | ND | ND | ND | ND | ND | ND | ND | ND | ND | K. Thaler | / |
| **HNHM 12678** | Chiavon | Vicenza | VI | Veneto | Italy | ND | ND | ND | 20/09/1970 | ND | ND | ND | ND | ND | ND | ND | ND | ND | ND | ND | ND | ND | ND | ND | ND | ND | ND | ND | Caobelli | / |
