# Supplementary material for: Barcoding Eophila crodabepis sp. nov. (Annelida, Oligochaeta, Lumbricidae), a Large Stripy Earthworm from Alpine Foothills of Northeastern Italy Similar to Eophila tellinii (Rosa, 1888)
Source: PLoS One. 2016 Mar 28;11(3):e0151799. doi: 10.1371/journal.pone.0151799 (PMC4809493; doi:10.1371/journal.pone.0151799)
Supplement: S2 Table — (DOC) [file pone.0151799.s005.doc]

| **SAMPLE** | **LIFE STAGE** | **PROSTOMIUM** | **SETAE** | **CLITELLUM** | **CLITELLUM SEGMENTS** | **TUBERCULA PUBERTATIS** | **aa** | **ab** | **bc** | **cd** | **dd** | **U** | **Fixed specimen height (cm) at the clitellum or in the highest part of the body (in imm.s)** | **TOTAL SEGMENTS NUMBER** | **PIGMENT** | **Dry specimen AVERAGE WEIGHT (gr.)** | **Fixed specimen LENGHT (cm)** | **BODY'S SHAPE** | **Fixed specimen diameter at the clitellum or in the thickest part of the body (in imm.s) (cm)** | **First dorsal pore** | **Male pores** | **SIZE Male pores** | **Female pores** | **SETAL PAPILLAE** | **FIRST PAIR OF NEPHRIDIA** | **SPERMATHECAE** | **SPERMATHECAL PORES** | **SEMINAL VESICLES** | **TYPHLOSOLE** | **GIZZARD (segments)** | **TESTES (segments)** | **SEMINIFEROUS FUNNELS (segments)** | **OVARIES (segments)** | **OVISACS (segments)** | **CALCIFEROUS GLANDS (segments)** | **CROP (segments)** | **HEARTS** | **COLLECTION NOTE** | **ANATOMIC DISSECTION** | **MICRO COMPUTED TOMOGRAPHY** | **DNA-BARCODING** | **REFERENCES** |
| --- | --- | --- | --- | --- | --- | --- | --- | --- | --- | --- | --- | --- | --- | --- | --- | --- | --- | --- | --- | --- | --- | --- | --- | --- | --- | --- | --- | --- | --- | --- | --- | --- | --- | --- | --- | --- | --- | --- | --- | --- | --- | --- |
| Toppo Carnico 1 | ad. | epil. | closely paired | 28-43 | 16 | 32-39 | 13.3 | 1.33 | 7.5 | 1 | 28.5 | 61.5 | ND | 324 | ND | 32.3 | ND | ND | ND | F 5/6 | ND | ND | ND | ND | ND | ND | ND | ND | ND | ND | ND | ND | ND | ND | ND | ND | ND | ND | NO | NO | NO | Braido. 1993 |
| Toppo Carnico 2 | ad. | epil. | closely paired | 28-43 | 16 | 32-39 | 13.3 | 1.33 | 7.5 | 1 | 28.5 | 61.5 | ND | 367 | ND | 39.7 | ND | ND | ND | F 5/6 | ND | ND | ND | ND | ND | ND | ND | ND | ND | ND | ND | ND | ND | ND | ND | ND | ND | ND | NO | NO | NO | Braido. 1993 |
| Toppo Carnico 3 | ad. | epil. | closely paired | 28-43 | 16 | ND | 13.3 | 1.33 | 7.5 | 1 | 28.5 | 61.5 | ND | 315 | ND | ND | ND | ND | ND | F 5/6 | ND | ND | ND | ND | ND | ND | ND | ND | ND | ND | ND | ND | ND | ND | ND | ND | ND | ND | NO | NO | NO | Braido. 1993 |
| Toppo Carnico 4 | ad. | epil. | closely paired | 27-43 | 17 | 33-41 | 13.3 | 1.33 | 7.5 | 1 | 28.5 | 61.5 | ND | ND | ND | ND | ND | ND | ND | F 5/6 | ND | ND | ND | ND | ND | ND | ND | ND | ND | ND | ND | ND | ND | ND | ND | ND | ND | ND | NO | NO | NO | Braido. 1993 |
| Toppo Carnico 5 | ad. | epil. | closely paired | 27-43 | 17 | 32-39 | 13.3 | 1.33 | 7.5 | 1 | 28.5 | 61.5 | ND | 324 | ND | 33.5 | ND | ND | ND | F 5/6 | ND | ND | ND | ND | ND | ND | ND | ND | ND | ND | ND | ND | ND | ND | ND | ND | ND | ND | NO | NO | NO | Braido. 1993 |
| Toppo Carnico 6 | ad. | epil. | closely paired | 27-43 | 17 | 32-39 | 13.3 | 1.33 | 7.5 | 1 | 28.5 | 61.5 | ND | 328 | ND | 43.5 | ND | ND | ND | F 5/6 | ND | ND | ND | ND | ND | ND | ND | ND | ND | ND | ND | ND | ND | ND | ND | ND | ND | ND | NO | NO | NO | Braido. 1993 |
| Toppo Carnico 7 | imm. | epil. | ND | / | / | / | ND | ND | ND | ND | ND | ND | ND | 354 | ND | 2.68 | ND | ND | ND | ND | ND | ND | ND | ND | ND | ND | ND | ND | ND | ND | ND | ND | ND | ND | ND | ND | ND | ND | NO | NO | NO | Braido. 1993 |
| Toppo Carnico 8 | imm. | epil. | ND | / | / | / | ND | ND | ND | ND | ND | ND | ND | 324 | ND | 2.68 | ND | ND | ND | ND | ND | ND | ND | ND | ND | ND | ND | ND | ND | ND | ND | ND | ND | ND | ND | ND | ND | ND | NO | NO | NO | Braido. 1993 |
| Toppo Carnico 9 | imm. | epil. | ND | / | / | / | ND | ND | ND | ND | ND | ND | ND | 324 | ND | 5.14 | ND | ND | ND | ND | ND | ND | ND | ND | ND | ND | ND | ND | ND | ND | ND | ND | ND | ND | ND | ND | ND | ND | NO | NO | NO | Braido. 1993 |
| Toppo Carnico 10 | imm. | epil. | ND | / | / | / | ND | ND | ND | ND | ND | ND | ND | 361 | ND | 3.67 | ND | ND | ND | ND | ND | ND | ND | ND | ND | ND | ND | ND | ND | ND | ND | ND | ND | ND | ND | ND | ND | ND | NO | NO | NO | Braido. 1993 |
| Toppo Carnico 11 | imm. | epil. | ND | / | / | / | ND | ND | ND | ND | ND | ND | ND | 335 | ND | 6.11 | ND | ND | ND | ND | ND | ND | ND | ND | ND | ND | ND | ND | ND | ND | ND | ND | ND | ND | ND | ND | ND | ND | NO | NO | NO | Braido. 1993 |
| Toppo Carnico 12 | imm. | epil. | ND | / | / | / | ND | ND | ND | ND | ND | ND | ND | 371 | ND | ND | ND | ND | ND | ND | ND | ND | ND | ND | ND | ND | ND | ND | ND | ND | ND | ND | ND | ND | ND | ND | ND | ND | NO | NO | NO | Braido. 1993 |
| Toppo Carnico 13 | imm. | epil. | ND | / | / | / | ND | ND | ND | ND | ND | ND | ND | 324 | ND | 14.7 | ND | ND | ND | ND | ND | ND | ND | ND | ND | ND | ND | ND | ND | ND | ND | ND | ND | ND | ND | ND | ND | ND | NO | NO | NO | Braido. 1993 |
| Toppo Carnico 14 | imm. | epil. | ND | / | / | / | ND | ND | ND | ND | ND | ND | ND | 364 | ND | 15.5 | ND | ND | ND | ND | ND | ND | ND | ND | ND | ND | ND | ND | ND | ND | ND | ND | ND | ND | ND | ND | ND | ND | NO | NO | NO | Braido. 1993 |
| Toppo Carnico 15 | imm. | epil. | ND | / | / | / | ND | ND | ND | ND | ND | ND | ND | 356 | ND | 4.14 | ND | ND | ND | ND | ND | ND | ND | ND | ND | ND | ND | ND | ND | ND | ND | ND | ND | ND | ND | ND | ND | ND | NO | NO | NO | Braido. 1993 |
| Toppo Carnico 16 | imm. | epil. | ND | / | / | / | ND | ND | ND | ND | ND | ND | ND | 211 | ND | 16.7 | ND | ND | ND | ND | ND | ND | ND | ND | ND | ND | ND | ND | ND | ND | ND | ND | ND | ND | ND | ND | ND | ND | NO | NO | NO | Braido. 1993 |
| Toppo Carnico 17 | imm. | epil. | ND | / | / | / | ND | ND | ND | ND | ND | ND | ND | 371 | ND | 1.83 | ND | ND | ND | ND | ND | ND | ND | ND | ND | ND | ND | ND | ND | ND | ND | ND | ND | ND | ND | ND | ND | ND | NO | NO | NO | Braido. 1993 |
| Toppo Carnico 18 | imm. | epil. | ND | / | / | / | ND | ND | ND | ND | ND | ND | ND | 327 | ND | 6.21 | ND | ND | ND | ND | ND | ND | ND | ND | ND | ND | ND | ND | ND | ND | ND | ND | ND | ND | ND | ND | ND | ND | NO | NO | NO | Braido. 1993 |
| Toppo Carnico 19 | imm. | epil. | ND | / | / | / | ND | ND | ND | ND | ND | ND | ND | 316 | ND | 2.23 | ND | ND | ND | ND | ND | ND | ND | ND | ND | ND | ND | ND | ND | ND | ND | ND | ND | ND | ND | ND | ND | ND | NO | NO | NO | Braido. 1993 |
| Toppo Carnico 20 | imm. | epil. | ND | / | / | / | ND | ND | ND | ND | ND | ND | ND | 327 | ND | 2.51 | ND | ND | ND | ND | ND | ND | ND | ND | ND | ND | ND | ND | ND | ND | ND | ND | ND | ND | ND | ND | ND | ND | NO | NO | NO | Braido. 1993 |
| Toppo Carnico 21 | imm. | epil. | ND | / | / | / | ND | ND | ND | ND | ND | ND | ND | 354 | ND | 2.28 | ND | ND | ND | ND | ND | ND | ND | ND | ND | ND | ND | ND | ND | ND | ND | ND | ND | ND | ND | ND | ND | ND | NO | NO | NO | Braido. 1993 |
| Toppo Carnico 22 | imm. | epil. | ND | / | / | / | ND | ND | ND | ND | ND | ND | ND | 374 | ND | 3.28 | ND | ND | ND | ND | ND | ND | ND | ND | ND | ND | ND | ND | ND | ND | ND | ND | ND | ND | ND | ND | ND | ND | NO | NO | NO | Braido. 1993 |
| Toppo Carnico 23 | imm. | epil. | ND | / | / | / | ND | ND | ND | ND | ND | ND | ND | 365 | ND | 11.7 | ND | ND | ND | ND | ND | ND | ND | ND | ND | ND | ND | ND | ND | ND | ND | ND | ND | ND | ND | ND | ND | ND | NO | NO | NO | Braido. 1993 |
| Borgo Cilia 1 | ad. | epil. | closely paired | 28-43 | 16 | 30-39 | 13.3 | 1.33 | 7.5 | 1 | 28.5 | 61.5 | ND | 331 | ND | 24.6 | ND | ND | ND | F 5/6 | ND | ND | ND | ND | ND | ND | ND | ND | ND | ND | ND | ND | ND | ND | ND | ND | ND | ND | NO | NO | NO | Braido. 1993 |
| Borgo Cilia 2 | ad. | epil. | closely paired | 28-43 | 16 | 30-39 | 13.3 | 1.33 | 7.5 | 1 | 28.5 | 61.5 | ND | 335 | ND | 32.9 | ND | ND | ND | F 5/6 | ND | ND | ND | ND | ND | ND | ND | ND | ND | ND | ND | ND | ND | ND | ND | ND | ND | 34** | NO | NO | NO | Braido. 1993 |
| Borgo Cilia 3 | ad. | epil. | closely paired | 29-43 | 15 | ND | 13.3 | 1.33 | 7.5 | 1 | 28.5 | 61.5 | ND | 330 | ND | 18.6 | ND | ND | ND | F 5/6 | ND | ND | ND | ND | ND | ND | ND | ND | ND | ND | ND | ND | ND | ND | ND | ND | ND | ND | NO | NO | NO | Braido. 1993 |
| Borgo Cilia 4 | ad. | epil. | closely paired | 28-43 | 16 | 30-39 | 13.3 | 1.33 | 7.5 | 1 | 28.5 | 61.5 | ND | 354 | ND | 29.7 | ND | ND | ND | F 5/6 | ND | ND | ND | ND | ND | ND | ND | ND | ND | ND | ND | ND | ND | ND | ND | ND | ND | ND | NO | NO | NO | Braido. 1993 |
| Borgo Cilia 5 | ad. | epil. | closely paired | 27-43 | 17 | 30-39 | 13.3 | 1.33 | 7.5 | 1 | 28.5 | 61.5 | ND | 343 | ND | 19.5 | ND | ND | ND | F 5/6 | ND | ND | ND | ND | ND | ND | ND | ND | ND | ND | ND | ND | ND | ND | ND | ND | ND | ND | NO | NO | NO | Braido. 1993 |
| Borgo Cilia 6 | ad. | epil. | closely paired | 27-43 | 17 | 28-40 | 13.3 | 1.33 | 7.5 | 1 | 28.5 | 61.5 | ND | 300 | ND | 38 | ND | ND | ND | F 5/6 | ND | ND | ND | ND | ND | ND | ND | ND | ND | ND | ND | ND | ND | ND | ND | ND | ND | 175** | NO | NO | NO | Braido. 1993 |
| Borgo Cilia 7 | ad. | epil. | closely paired | 29-43 | 15 | 28-40 | 13.3 | 1.33 | 7.5 | 1 | 28.5 | 61.5 | ND | 317 | ND | 41.9 | ND | ND | ND | F 5/6 | ND | ND | ND | ND | ND | ND | ND | ND | ND | ND | ND | ND | ND | ND | ND | ND | ND | 71** | NO | NO | NO | Braido. 1993 |
| Borgo Cilia 8 | imm. | epil. | ND | / | / | / | ND | ND | ND | ND | ND | ND | ND | 329 | ND | 36.7 | ND | ND | ND | ND | ND | ND | ND | ND | ND | ND | ND | ND | ND | ND | ND | ND | ND | ND | ND | ND | ND | ND | NO | NO | NO | Braido. 1993 |
| Borgo Cilia 9 | imm. | epil. | ND | / | / | / | ND | ND | ND | ND | ND | ND | ND | 319 | ND | 15.5 | ND | ND | ND | ND | ND | ND | ND | ND | ND | ND | ND | ND | ND | ND | ND | ND | ND | ND | ND | ND | ND | ND | NO | NO | NO | Braido. 1993 |
| Borgo Cilia 10 | imm. | epil. | ND | / | / | / | ND | ND | ND | ND | ND | ND | ND | 340 | ND | 17.8 | ND | ND | ND | ND | ND | ND | ND | ND | ND | ND | ND | ND | ND | ND | ND | ND | ND | ND | ND | ND | ND | ND | NO | NO | NO | Braido. 1993 |
| Borgo Cilia 11 | imm. | epil. | ND | / | / | / | ND | ND | ND | ND | ND | ND | ND | 341 | ND | 2.82 | ND | ND | ND | ND | ND | ND | ND | ND | ND | ND | ND | ND | ND | ND | ND | ND | ND | ND | ND | ND | ND | ND | NO | NO | NO | Braido. 1993 |
| Follina 1 | ad. | epil. | closely paired | 25-37 | 13 | ND | 13.3 | 1.33 | 7.5 | 1 | 28.5 | 61.5 | ND | 264 | ND | 21.7 | ND | ND | ND | F 5/6 | ND | ND | ND | ND | ND | ND | ND | ND | ND | ND | ND | ND | ND | ND | ND | ND | ND | ND | NO | NO | NO | Braido. 1993 |
| Follina 2 | ad. | epil. | closely paired | 25-37 | 13 | 28-39 | 13.3 | 1.33 | 7.5 | 1 | 28.5 | 61.5 | ND | 262 | ND | 15.3 | ND | ND | ND | F 5/6 | ND | ND | ND | ND | ND | ND | ND | ND | ND | ND | ND | ND | ND | ND | ND | ND | ND | 71** | NO | NO | NO | Braido. 1993 |
| Follina 3 | ad. | epil. | closely paired | 25-37 | 13 | 29-36 | 13.3 | 1.33 | 7.5 | 1 | 28.5 | 61.5 | ND | 284 | ND | 22.7 | ND | ND | ND | F 5/6 | ND | ND | ND | ND | ND | ND | ND | ND | ND | ND | ND | ND | ND | ND | ND | ND | ND | ND | NO | NO | NO | Braido. 1993 |
| Follina 4 | ad. | epil. | closely paired | 25-37 | 13 | 29-36 | 13.3 | 1.33 | 7.5 | 1 | 28.5 | 61.5 | ND | 272 | ND | 15 | ND | ND | ND | F 5/6 | ND | ND | ND | ND | ND | ND | ND | ND | ND | ND | ND | ND | ND | ND | ND | ND | ND | ND | NO | NO | NO | Braido. 1993 |
| Follina 5 | ad. | epil. | closely paired | 25-37 | 13 | 29-36 | 13.3 | 1.33 | 7.5 | 1 | 28.5 | 61.5 | ND | 254 | ND | 20.1 | ND | ND | ND | F 5/6 | ND | ND | ND | ND | ND | ND | ND | ND | ND | ND | ND | ND | ND | ND | ND | ND | ND | ND | NO | NO | NO | Braido. 1993 |
| Follina 6 | ad. | epil. | closely paired | 25-37 | 13 | 29-36 | 13.3 | 1.33 | 7.5 | 1 | 28.5 | 61.5 | ND | 246 | ND | 17.3 | ND | ND | ND | F 5/6 | ND | ND | ND | ND | ND | ND | ND | ND | ND | ND | ND | ND | ND | ND | ND | ND | ND | ND | NO | NO | NO | Braido. 1993 |
| Follina 7 | imm. | epil. | ND | / | / | / | ND | ND | ND | ND | ND | ND | ND | 264 | ND | 0.82 | ND | ND | ND | ND | ND | ND | ND | ND | ND | ND | ND | ND | ND | ND | ND | ND | ND | ND | ND | ND | ND | ND | NO | NO | NO | Braido. 1993 |
| Ciaurlec Mount 1 | ad. | epil. | closely paired | 27-42 | 16 | 31-38 | 13.3 | 1.33 | 7.5 | 1 | 28.5 | 61.5 | ND | 375 | ND | 49 | ND | ND | ND | F 5/6 | ND | ND | ND | ND | ND | ND | ND | ND | ND | ND | ND | ND | ND | ND | ND | ND | ND | ND | NO | NO | NO | Braido. 1993 |
| Ciaurlec Mount 2 | ad. | epil. | closely paired | 27-43 | 16 | 31-40 | 13.3 | 1.33 | 7.5 | 1 | 28.5 | 61.5 | ND | 325 | ND | 42.7 | ND | ND | ND | F 5/6 | ND | ND | ND | ND | ND | ND | ND | ND | ND | ND | ND | ND | ND | ND | ND | ND | ND | ND | NO | NO | NO | Braido. 1993 |
| Ciaurlec Mount 3 | imm. | epil. | ND | / | / | / | ND | ND | ND | ND | ND | ND | ND | 349 | ND | 4.95 | ND | ND | ND | ND | ND | ND | ND | ND | ND | ND | ND | ND | ND | ND | ND | ND | ND | ND | ND | ND | ND | ND | NO | NO | NO | Braido. 1993 |
| Ciaurlec Mount 4 | imm | pil | ND | / | / | / | ND | ND | ND | ND | ND | ND | ND | 272 | ND | 1.53 | ND | ND | ND | ND | ND | ND | ND | ND | ND | ND | ND | ND | ND | ND | ND | ND | ND | ND | ND | ND | ND | Predated | ND | ND | ND | Braido. 1993 |
| Tavaran Grande 1 | ad. | epil. | closely paired | 25-37 | 13 | 28-36 | 13.3 | 1.33 | 7.5 | 1 | 28.5 | 61.5 | ND | 187 | ND | 18.2 | ND | ND | ND | F 5/6 | ND | ND | ND | ND | ND | ND | ND | ND | ND | ND | ND | ND | ND | ND | ND | ND | ND | predated | NO | NO | NO | Braido. 1993 |
| Tavaran Grande 2 | imm. | epil. | ND | / | / | / | ND | ND | ND | ND | ND | ND | ND | 212 | ND | 2.18 | ND | ND | ND | ND | ND | ND | ND | ND | ND | ND | ND | ND | ND | ND | ND | ND | ND | ND | ND | ND | ND | ND | NO | NO | NO | Braido. 1993 |
| Tavaran Grande 3 | imm. | epil. | ND | / | / | / | ND | ND | ND | ND | ND | ND | ND | 265 | ND | 0.87 | ND | ND | ND | ND | ND | ND | ND | ND | ND | ND | ND | ND | ND | ND | ND | ND | ND | ND | ND | ND | ND | ND | NO | NO | NO | Braido. 1993 |
| Tavaran Grande 4 | imm. | epil. | ND | / | / | / | ND | ND | ND | ND | ND | ND | ND | 263 | ND | 5.53 | ND | ND | ND | ND | ND | ND | ND | ND | ND | ND | ND | ND | ND | ND | ND | ND | ND | ND | ND | ND | ND | ND | NO | NO | NO | Braido. 1993 |
| Tavaran Grande 5 | imm. | epil. | ND | / | / | / | ND | ND | ND | ND | ND | ND | ND | 214 | ND | 1.54 | ND | ND | ND | ND | ND | ND | ND | ND | ND | ND | ND | ND | ND | ND | ND | ND | ND | ND | ND | ND | ND | ND | NO | NO | NO | Braido. 1993 |
| Tavaran Grande 6 | imm. | epil. | ND | / | / | / | ND | ND | ND | ND | ND | ND | ND | 253 | ND | 2.25 | ND | ND | ND | ND | ND | ND | ND | ND | ND | ND | ND | ND | ND | ND | ND | ND | ND | ND | ND | ND | ND | ND | NO | NO | NO | Braido. 1993 |
| Tavaran Grande 7 | imm. | epil. | ND | / | / | / | ND | ND | ND | ND | ND | ND | ND | 250 | ND | 2 | ND | ND | ND | ND | ND | ND | ND | ND | ND | ND | ND | ND | ND | ND | ND | ND | ND | ND | ND | ND | ND | ND | NO | NO | NO | Braido. 1993 |
| Altopiano d'Asiago 1 *** | ad. | 1/3 epil.. closed | closely paired? | ½ 25-38 | 14 | 29-35 (right) and 31-37 (left) | 13.3 *** | 1.33 | 7.5 | 1 | 28.5 | 61.5 | ND | ND | bands of purplish-brown pigment are present around the middle of each segment.. | 17.1 | 26 | almost cylindrical. but the posterior end can be somewhat swollen | 1.5 | F 5/6 | 15 | with slightly swollen lips. with a small porophore. between setae b and c | 14 near setae b | ab on segments 9-13 | 6 segment | 2 pairs? | Fs 9/10 and 10/11 along the lines of setae cd | 4 pairs. in the 9-10-11-12 segments | begins in the region of the clitellum and has the form of a cord with two longitudinal ridges | 17-19 | in usual site | in usual site | in usual position | in usual position | 10 | 15 and 16 | 6 pairs. in segments 6-11 | ND | YES | NO | NO | Omodeo. 1988 |
| Altopiano d'Asiago 2 *** | imm. | 1/3 epil.. closed | closely paired? | / | / | / | ND | ND | ND | ND | ND | ND | ND | ND | bands of purplish-brown pigment are present around the middle of each segment.. | ND | ND | ND | ND | F 5/6 | 15 | with slightly swollen lips. with a small porophore. between setae b and c | 14 near setae b | ab on segments 9-13 | ND | ND | ND | ND | ND | ND | ND | ND | ND | ND | ND | ND | ND | ND | NO | NO | NO | Omodeo. 1988 |
| Valdobbiadene 1 *** | subad. | 1/3 epil.. closed | closely paired? | ND | 13 | ND | 7.5 | 1.1 | 5.5 | 1 | 27 | 47 | ND | ND | darker than other Eophila tellinii found in Altopiano d'Asiago. bands of purplish-brown pigment are present around the middle of each segment.. | ND | ND | ND | ND | F 5/6 | 15 | with slightly swollen lips. with a small porophore. between setae b and c | 14 near setae b | ab on segments 9-13 | 6 segment | 2 pairs? | Fs 9/10 and 10/11 along the lines of setae cd | 4 pairs. in the 9-10-11-12 segments | begins in the region of the clitellum and has the form of a cord with two longitudinal ridges | 17-19 | in usual site |  | in usual position | in usual position | 10 | 15 and 16 | 6 pairs. in segments 6-11 | ND | YES | NO | NO | Omodeo. 1988 |
| Valdobbiadene 2 *** | imm. | 1/3 epil.. closed | closely paired? | / | / | / | ND | ND | ND | ND | ND | ND | ND | ND | darker than other Eophila tellinii found in Altopiano d'Asiago. bands of purplish-brown pigment are present around the middle of each segment.. | ND | ND | ND | ND | F 5/6 | 15 | with slightly swollen lips. with a small porophore. between setae b and c | 14 near setae b | ab on segments 9-13 | ND | ND | ND | ND | ND | ND | ND | ND | ND | ND | ND | ND | ND | ND | NO | NO | NO | Omodeo. 1988 |
| Val Posan 10 | ad. | epil. | closely paired | 25-39 | 15 | ND | 13.3 | 1.33 | 7.5 | 1 | 28.5 | 61.5 | ND | 154 | ND | 13.3 | ND | ND | ND | F 5/6 | ND | ND | ND | ND | ND | ND | ND | ND | ND | ND | ND | ND | ND | ND | ND | ND | ND | predated | NO | NO | NO | Braido. 1993 |
| Val Posan 11 | imm. | epil. | ND | / | / | / | ND | ND | ND | ND | ND | ND | ND | 264 | ND | 3.39 | ND | ND | ND | ND | ND | ND | ND | ND | ND | ND | ND | ND | ND | ND | ND | ND | ND | ND | ND | ND | ND | ND | NO | NO | NO | Braido. 1993 |
| Val Posan 12 | imm. | epil. | ND | / | / | / | ND | ND | ND | ND | ND | ND | ND | 220 | ND | 2.71 | ND | ND | ND | ND | ND | ND | ND | ND | ND | ND | ND | ND | ND | ND | ND | ND | ND | ND | ND | ND | ND | ND | NO | NO | NO | Braido. 1993 |
| Val Posan 13 | imm. | epil. | ND | / | / | / | ND | ND | ND | ND | ND | ND | ND | 264 | ND | 1.29 | ND | ND | ND | ND | ND | ND | ND | ND | ND | ND | ND | ND | ND | ND | ND | ND | ND | ND | ND | ND | ND | ND | NO | NO | NO | Braido. 1993 |
| Val Posan 14 | imm. | epil. | ND | / | / | / | ND | ND | ND | ND | ND | ND | ND | 280 | ND | 1.27 | ND | ND | ND | ND | ND | ND | ND | ND | ND | ND | ND | ND | ND | ND | ND | ND | ND | ND | ND | ND | ND | ND | NO | NO | NO | Braido. 1993 |
| S. Boldo Pass *** | subad. | 1/3 epil.. closed | closely paired? | ND | 13 | ND | 7.5 | 1.1 | 5.5 | 1 | 27 | 47 | ND | ND | darker than other Eophila tellinii found in Altopiano d'Asiago. bands of purplish-brown pigment are present around the middle of each segment.. | ND | ND | ND | ND | F 5/6 | 15 | with slightly swollen lips. with a small porophore. between setae b and c | 14 near setae b | ab on segments 9-13 | 6 segment | 2 pairs? | Fs 9/10 and 10/11 along the lines of setae cd | 4 pairs. in the 9-10-11-12 segments | begins in the region of the clitellum and has the form of a cord with two longitudinal ridges | 17-19 | in usual site |  | in usual position | in usual position | 10 | 15-16 | 6 pairs. in segments 6-11 | ND | YES | NO | NO | Omodeo. 1988 |

**S2 Table. Morphological and anatomical feautures of the specimens taken from the literature.**

(post clitellar setal ratio. ab=1); ** = last segments regenerated; *** = setal ratio related to segment 50
