# Supplementary material for: Barcoding Eophila crodabepis sp. nov. (Annelida, Oligochaeta, Lumbricidae), a Large Stripy Earthworm from Alpine Foothills of Northeastern Italy Similar to Eophila tellinii (Rosa, 1888)
Source: PLoS One. 2016 Mar 28;11(3):e0151799. doi: 10.1371/journal.pone.0151799 (PMC4809493; doi:10.1371/journal.pone.0151799)
Supplement: S3 Table — (DOC) [file pone.0151799.s006.doc]

| **SAMPLE** | **LIFE STAGE** | **CLITELLUM** | **CLITELLUM SEGMENTS** | **TUBERCULA PUBERTATIS** | **TOTAL SEGMENTS NUMBER** | **Dry specimen AVERAGE WEIGHT (gr.)** | **Fixed specimen LENGHT (mm)** | **Fixed specimen diameter at the clitellum or in the thickest part of the body (in imm.s) (mm)** | **First dorsal pore** | **SETAL PAPILLAE** | **FIRST PAIR OF NEPHRIDIA** | **SPERMATHECAE (segments)** | **SPERMATHECAL PORES** | **SEMINAL VESICLES** | **GIZZARD (segments)** | **TESTES (segments)** | **SEMINIFEROUS FUNNELS (segments)** | **OVARIES (segments)** | **OVISACS (segments)** | **CALCIFEROUS GLANDS (segments)** | **CROP (segments)** | **HEARTS** | **COLLECTION NOTE** | **ANATOMIC DISSECTION** | **MICRO COMPUTED TOMOGRAPHY** | **DNA-BARCODING** | **REFERENCES** |
| --- | --- | --- | --- | --- | --- | --- | --- | --- | --- | --- | --- | --- | --- | --- | --- | --- | --- | --- | --- | --- | --- | --- | --- | --- | --- | --- | --- |
| **Ragogna 1** | ad. | 27-41 | 15 | 31-37 | 276 | 13.6 | 210 | 10 | 5/6 | 8-11 | 4 | 10.11 | 9/10-10/11 | *** | 17-19 | 10-11 | 10-11 | 13 | 14 | 10-12 | 15-16 | 6-11 | 25-26* | YES | NO | 2013 | / |
| **Ragogna 2** | ad. | 27-40 | 14 | 31-37 | 277 | 16 | 250 | 10 | 5/6 | / | / | ND | ND | ND | ND | ND | ND | ND | ND | ND | ND | ND | 25-26* | NO | YES | 2013 | / |
| **Ragogna 3** | ad. | 27-41 | 15 | 31-37 | 283 | 11.4 | 230 | 12 | 5/6 | 8-11 | ND | ND | ND | / | / | / | / | / | / | / | / | / | 25-26* | YES | NO | 2013 | / |
| **Ragogna 4** | ad. | 27-41 | 15 | 31-37 | 250 | 11.9 | 170 | 11 | 5/6 | 7-12 | ND | ND | ND | ND | ND | ND | ND | ND | ND | ND | ND | ND | 25-26* | YES | NO | 2013 | / |
| **Ragogna 5** | imm. | / | / | / | 261 | 0.84 | 75 | 4 | / | / | ND | ND | ND | / | / | / | / | / | / | / | / | / | / | NO | NO | 2013 | / |
| **Travesio 22** | ad. | 27-44 | 18 | 32-40 | 341 | 27.04 | 310 | 14 | F 5/6 | 6-13 | ND | ND | ND | / | / | / | / | / | / | / | / | / | / | NO | NO | 2012 | / |
| **Travesio 23** | ad. | 26-44 | 19 | 31-40 | 315 | 28.2 | 360 | 13 | F 5/6 | 7-13 | ND | ND | ND | / | / | / | / | / | / | / | / | / | / | NO | NO | 2012 | / |
| **Clauzetto 21** | ad. | 26-40 | 15 | 30-38 | 259 | 20.6 | 250 | 12 | F 5/6 | / | ND | ND | ND | / | / | / | / | / | / | / | / | / | 25 and 41* | NO | NO | 2012 | / |
| **Clauzetto 2** | ad. | 27-41 | 15 | 30-38 | 257 | ND | 280 | 11 | F 5/6 | 8-12 | / | ND | ND | ND | ND | ND | ND | ND | ND | ND | ND | ND | / | NO | YES | NO | / |
| **Fornace Toppo 1** | imm. | / | / | / | 275 | 2.01 | 180 | 6 | F 5/6 | / | ND | ND | ND | / | / | / | / | / | / | / | / | / | / | NO | NO | 2014 | / |
| **Fornace Toppo 2** | imm. | / | / | / | 193 | 2.53 | 130 | 8 | F 5/6 | / | ND | ND | ND | / | / | / | / | / | / | / | / | / | / | NO | NO | 2014 | / |
| **Ciaurlec Mount** | ad. | 27-42 | 16 | 32-39 | 319 | 22.03 | 280 | 11 | ND | 7-13 | ND | ND | ND | / | / | / | / | / | / | / | / | / | / | NO | NO | 2012 | / |
| **Pradis 1 **** | / | / | / | / | / | / | / | / | / | / | / | / | / | / | / | / | / | / | / | / | / | / | Not collected | NO | NO | NO | / |
| **Pradis 2 **** | / | / | / | / | / | / | / | / | / | / | / | / | / | / | / | / | / | / | / | / | / | / | Not collected | NO | NO | NO | / |
| **Campo Solagna 17** | ND | ND | ND | ND | ND | ND | ND | ND | ND | ND | ND | ND | ND | ND | ND | ND | ND | ND | ND | ND | ND | ND | ND | ND | ND | 2012 | / |
| **Casasola **** | / | / | / | / | / | / | / | / | / | / | / | / | / | / | / | / | / | / | / | / | / | / | Not collected | NO | NO | NO | / |
| **Cesen 6** | imm. | / | / | / | 232 | 1.25 | 87 | 5 | F 5/6 | / | ND | ND | ND | / | / | / | / | / | / | / | / | / | / | NO | NO | 2014 | / |
| **Crevada 1** | ad. | 25-37 | 13 | 30-36 | 219 | 2.64 | 120 | 8 | F 5/6 | / | ND | ND | ND | / | / | / | / | / | / | / | / | / | / | NO | NO | 2014 | / |
| **Crevada 2** | ad. | 24-37 | 14 | 30-36 | 220 | 2.58 | 110 | 8 | F 5/6 | / | ND | ND | ND | / | / | / | / | / | / | / | / | / | / | NO | NO | 2014 | / |
| **Crevada 3** | ad. | 25-37 | 13 | 30-36 | 209 | 5.07 | 130 | 10 | F 5/6 | / | ND | ND | ND | / | / | / | / | / | / | / | / | / | / | NO | NO | 2014 | / |
| **Crevada 4** | ad. | 26-37 | 12 | 30-36 | 191 | 4.93 | 140 | 9 | F 5/6 | / | / | ND | ND | ND | ND | ND | ND | ND | ND | ND | ND | ND | 24-25* | YES | NO | 2014 | / |
| **Crevada 5** | ad. | 25-37 | 13 | 30-36 | 214 | 6.04 | 140 | 8 | F 5/6 | 8-13 | ND | ND | ND | / | / | / | / | / | / | / | / | / | / | NO | NO | NO | / |
| **Crevada 6** | ad. | ND | ND | ND | ND | ND | ND | ND | ND | ND | / | 10-11 | / | 9-12 | ND | ND | ND | ND | ND | ND | ND | 6-11 | / | NO | YES | NO | / |
| **Ekar 5** | ad. | 25-38 | 14 | ?30-36? | 250 | 10.71 | 190 | 12 | F 5/6 | / | ND | ND | ND | / | / | / | / | / | / | / | / | / | / | NO | NO | 2014 | / |
| **Fratte 50** | ad. | 26-37 | 12 | 30-36 | 248 | 5.94 | 180 | 9 | F 5/6 | 8-12 | ND | ND | ND | / | / | / | / | / | / | / | / | / | / | NO | NO | 2014 | / |
| **HNHM 6899** | Sub | 26–37 | 11 | 30–36 | / | / | / | / | / | / | / | / | / | / | / | / | / | / | / | / | / | / | / | / | / | / | / |
| **HNHM 12678** | ad | 26–38 | 12 | 1/29–36 | 260 | / | 240 | / | / | / | / | / | / | / | / | / | / | / | / | / | / | / | / | / | / | / | / |
| **Grappa Mount 1** | imm. | / | / | / | 267 | 8.07 | 210 | 8 | ND | / | ND | ND | ND | / | / | / | / | / | / | / | / | / | Predated | NO | NO | 2013 | / |
| **Grappa Mount 2** | ad. | 24-37 | 14 | 30-36 | 252 | 9.98 | 230 | 6 | F 5/6 | / | 4 | 10.11 | 9/10-10/11 | *** | 17-19 | 10-11 | 10-11 | 13 | 14 | 10-12 | 15-16 | 6-11 | / | YES | NO | 2013 | / |
| **Praderadego 24** | ad. | 26-37 | 12 | 30-36 | 221 | 7.75 | 180 | 8 | F 5/6 | / | ND | ND | ND | / | / | / | / | / | / | / | / | / | 24-25* | NO | NO | 2012 | / |
| **Praderadego 25** | ad. | 25-37 | 13 | 30-36 | 178 | 7.02 | 160 | 8 | F 5/6 | / | ND | ND | ND | / | / | / | / | / | / | / | / | / | / | NO | NO | 2012 | / |
| **Roncavezzai 1** | imm. | / | / | / | 233 | 2.34 | 150 | 8 | F 5/6 | / | ND | ND | ND | / | / | / | / | / | / | / | / | / | / | NO | NO | 2013 | / |
| **Roncavezzai 2** | ad. | 24-37 | 14 | 30-36 | 223 | 3.93 | 180 | 8 | F 5/6 | / | ND | ND | ND | / | / | / | / | / | / | / | / | / | / | NO | NO | 2013 | / |
| **Roncavezzai 3** | imm. | / | / | / | 173 | 0.59 | 60 | 6 | F 5/6 | / | ND | ND | ND | / | / | / | / | / | / | / | / | / | / | NO | NO | 2013 | / |
| **Roncavezzai 5** | ad. | 24-37 | 14 | 30-36 | 217 | 4.7 | 160 | 7 | F 5/6 | 8-12 | ND | ND | ND | / | / | / | / | / | / | / | / | / | / | NO | NO | 2012 | / |
| **Roncavezzai 6** | imm. | / | / | / | 235 | 1.75 | 150 | 5 | F 5/6 | / | ND | ND | ND | / | / | / | / | / | / | / | / | / | / | NO | NO | 2012 | / |
| **Roncavezzai 7** | imm. | / | / | / | 196 | 1.02 | 80 | 4 | F 5/6 | / | ND | ND | ND | / | / | / | / | / | / | / | / | / | / | NO | NO | NO | / |
| **Roncavezzai 8** | imm. | / | / | / | 232 | 1.35 | 130 | 4 | ND | / | ND | ND | ND | / | / | / | / | / | / | / | / | / | / | NO | NO | NO | / |
| **Roncavezzai 9** | imm. | / | / | / | 201 | 0.8 | 130 | 4 | ND | / | ND | ND | ND | / | / | / | / | / | / | / | / | / | / | NO | NO | 2012 | / |
| **Roncavezzai 10** | imm. | / | / | / | 132 | 0.27 | 63 | 3 | F 5/6 | / | ND | ND | ND | / | / | / | / | / | / | / | / | / | Predated | NO | NO | 2012 | / |
| **Roncavezzai 11** | ad. | 24-37 | 14 | 29-36 | 188 | 5.94 | 170 | 9 | F 5/6 | / | ND | ND | ND | / | / | / | / | / | / | / | / | / | Predated  23 and 38* | NO | NO | 2012 | / |
| **Roncavezzai 18** | ad. | 24-37 | 14 | 30-36 | 214 | 3.75 | 160 | 7 | ND | / | ND | ND | ND | / | / | / | / | / | / | / | / | / | / | NO | NO | 2012 | / |
| **Val Posan 1** | ad. | 25-37 | 13 | 30-36 | 146 | 12.5 | 220 | 9 | ND | 8-13 | ND | ND | ND | ND | ND | ND | ND | ND | ND | ND | ND | ND | ND | YES | NO | 2013 | / |
| **Val Posan 2** | ad. | 25-37 | 12 | 30-36 | 236 | 7.78 | 210 | 8 | F 5/6 | / | ND | ND | ND | / | / | / | / | / | / | / | / | / | Predated | YES | NO | 2013 | / |
| **Val Posan 3** | ad. | 24-36 | 13 | 30-36 | 139 | 4.75 | 100 | 9 | F 5/6 | / | ND | ND | ND | / | / | / | / | / | / | / | / | / | / | NO | NO | 2013 | / |
| **Val Posan 4** | ad. | 24-37 | 13 | 30-36 | 250 | 10.8 | 200 | 9 | ND | 8-12 | ND | ND | ND | / | / | / | / | / | / | / | / | / | / | NO | NO | 2013 | / |
| **Val Posan 5** | ad. | 24-36 | 13 | 30-36 | 242 | 5.18 | 150 | 9 | F 5/6 | / | ND | ND | ND | / | / | / | / | / | / | / | / | / | Predated | NO | NO | 2013 | / |
| **Val Posan 6** | imm. | / | / | / | 211 | 3.45 | 130 | 7 | F 5/6 | / | ND | ND | ND | / | / | / | / | / | / | / | / | / | / | NO | NO | 2013 | / |
| **Val Posan 7** | imm. | / | / | / | 142 | 1.52 | 84 | 5 | F 5/6 | / | ND | ND | ND | / | / | / | / | / | / | / | / | / | Predated | NO | NO | 2013 | / |
| **Val Posan 8** | imm. | / | / | / | 259 | 0.72 | 64 | 4 | F 5/6 | / | ND | ND | ND | / | / | / | / | / | / | / | / | / | Predated | NO | NO | NO | / |
| **Val Posan 9** | imm. | / | / | / | 232 | 0.7 | 76 | 4 | F 5/6 | / | ND | ND | ND | / | / | / | / | / | / | / | / | / | / | NO | NO | NO | / |
| **Villa Jacur 1** | imm. | / | / | / | 226 | 1.47 | 80 | 6 | F 5/6 | / | ND | ND | ND | / | / | / | / | / | / | / | / | / | / | NO | NO | 2013 | / |
| **Villa Jacur 2** | imm. | / | / | / | 184 | 0.17 | 43 | 4 | F 5/6 | / | ND | ND | ND | / | / | / | / | / | / | / | / | / | / | NO | NO | 2013 | / |
| **Villa Jacur 3** | imm. | / | / | / | 243 | 0.18 | 50 | 4 | F 5/6 | / | ND | ND | ND | / | / | / | / | / | / | / | / | / | / | NO | NO | 2013 | / |

**S3 Table. Morphology and anatomy of specimens inspected and from the literature.**

(Setal ratio in segment 12. ab=1)

* = segments already modified

** = specimens only observed

*** = 4 pairs. in the 9-10-11-12 segments

All specimens are epilobus with a closely paired setae and the males pores without tumescences. confined in 15 segment. between setae b and c. All specimens from Campo Solagna. Fornace Toppo. Monte Grappa. Pradis. Cassola. Roncavezzai. Val Posan and Villa Jacur there are no info about it.

All the bodies are cylindrical and depressed on the tail. the pigment of each specimens is purplish-brown banded in the middle of each segment All specimen form Campo Solagna. Pradis. Cassola and HNHM there are no info about it.

All specimens have male pore on 15th segment and female pore on 14th segment

(Ragogna 5; Fornace Toppo 1 and 2; Ciaurlec Mount; Pradis 1 and 2; Casasola; HNHM 6899; HNHM 12678; Roncavezzai 1. 3. 6 .7. 8. 9 and 10; Cesen 6; Val Posan 6. 7. 8 and 9; Villa Jacur 1. 2 and 3; No data available)

Typhlosole trifid on Ragogna 1 (23-212 segments). Crevada 6 (45 – 9 segments. from pygidium). Grappa Mount 2 (23-204 segments)
