# Supplementary figures and images for: Correction: Barcoding Eophila crodabepis sp. nov. (Annelida, Oligochaeta, Lumbricidae), a Large Stripy Earthworm from Alpine Foothills of Northeastern Italy Similar to Eophila tellinii (Rosa, 1888)
Source: PLoS One. 2016 Aug 1;11(8):e0160218. doi: 10.1371/journal.pone.0160218 (PMC4968801; doi:10.1371/journal.pone.0160218)

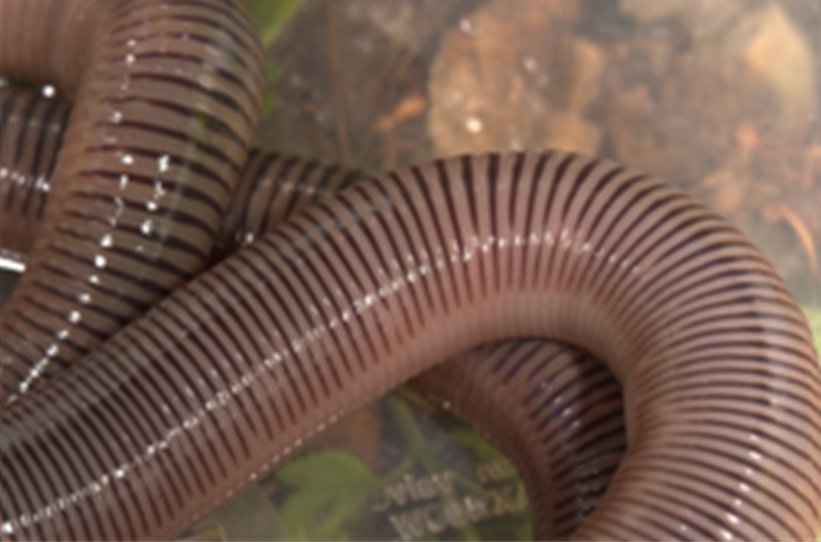


a)


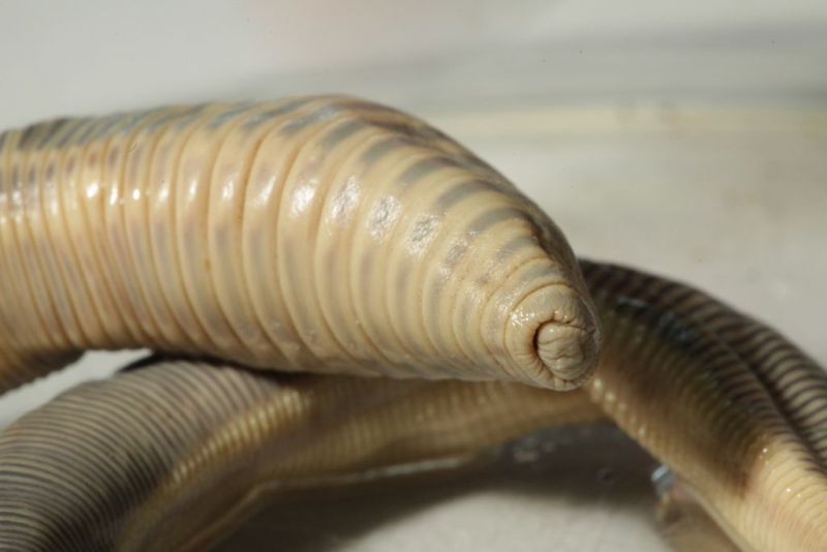


b)


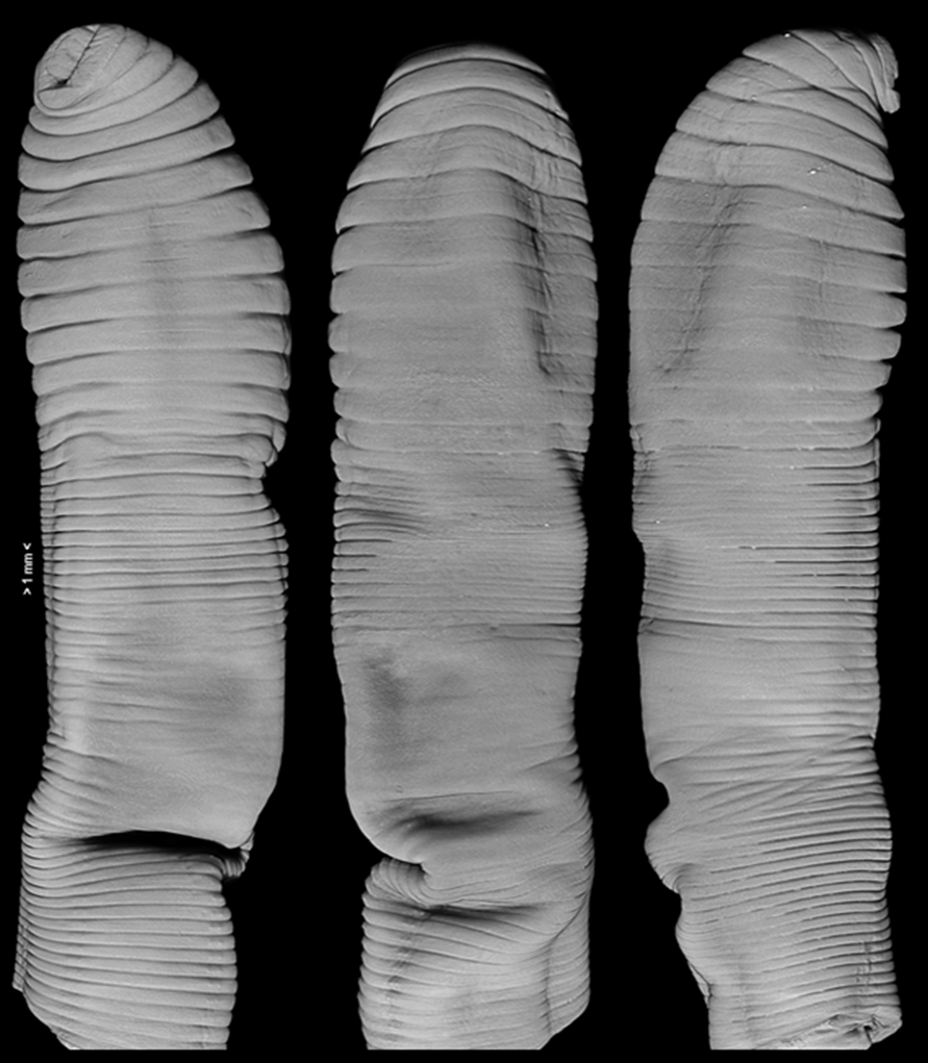


c)


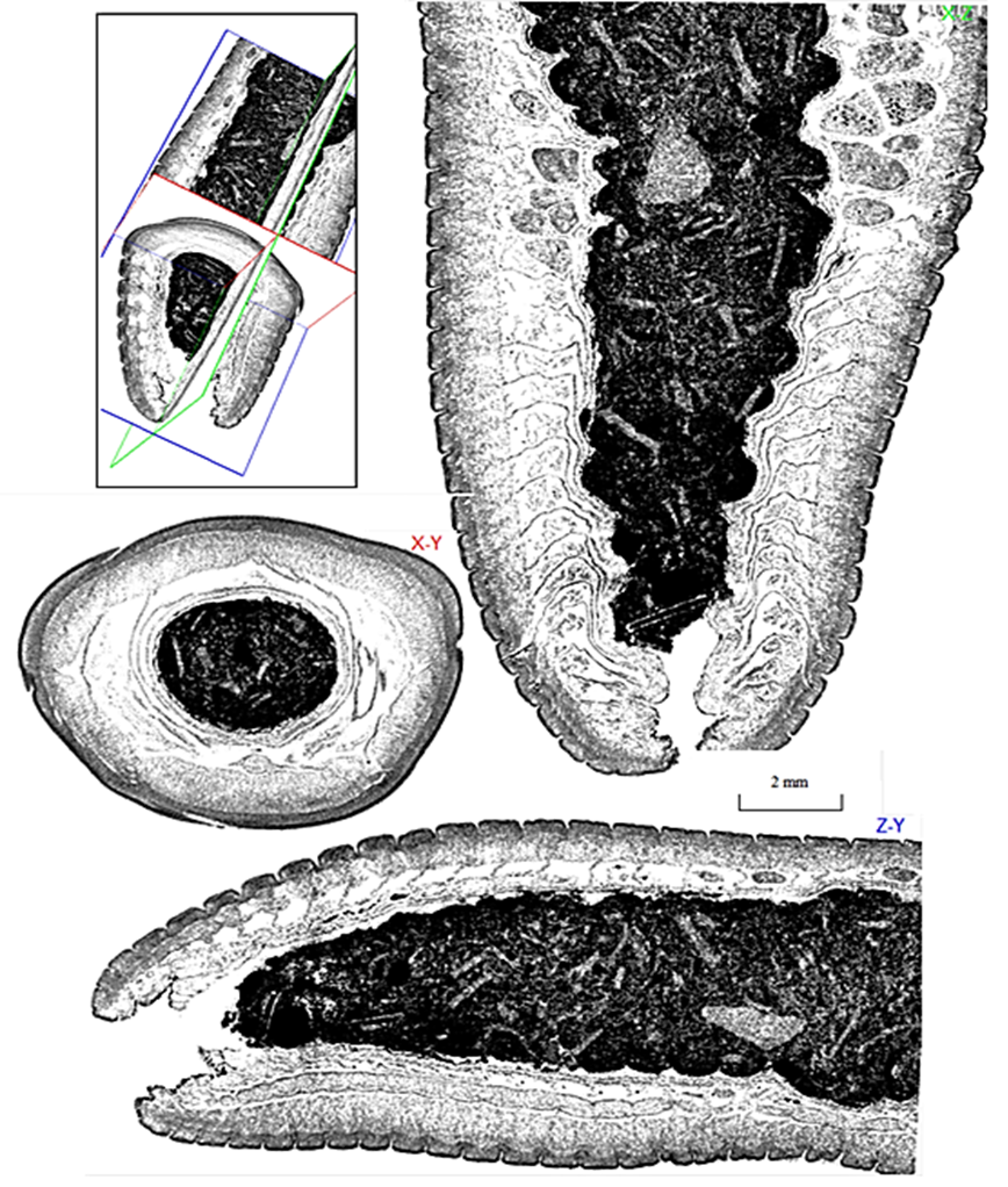


d)

Supplement: S1 Fig — a)Livery pattern in Travesio specimen and b) peristomial detail (Ragogna 1 specimen); c)7.98 µm and 4.35 µm ventral, dorsal and lateral views of specimen (Ragogna 2); d)virtual sections of hindmost segments: schematic and transversal sections of last ten segments lacking typhlosole, hindmost body segment- (middle) mesial-middle section (upper right), and sagittal medial section (bottom) from Clauzetto specimen. (DOC) [file pone.0160218.s001.doc]

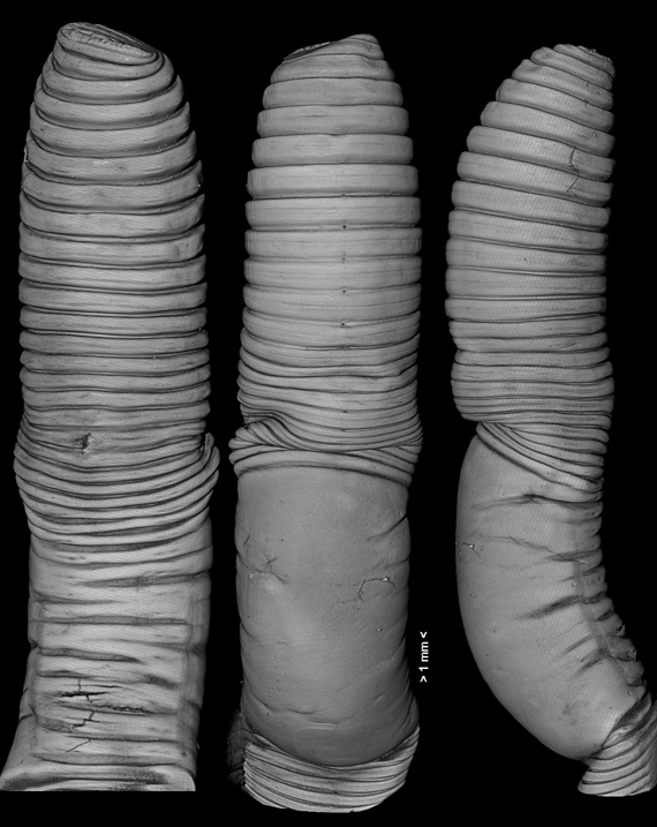


a)


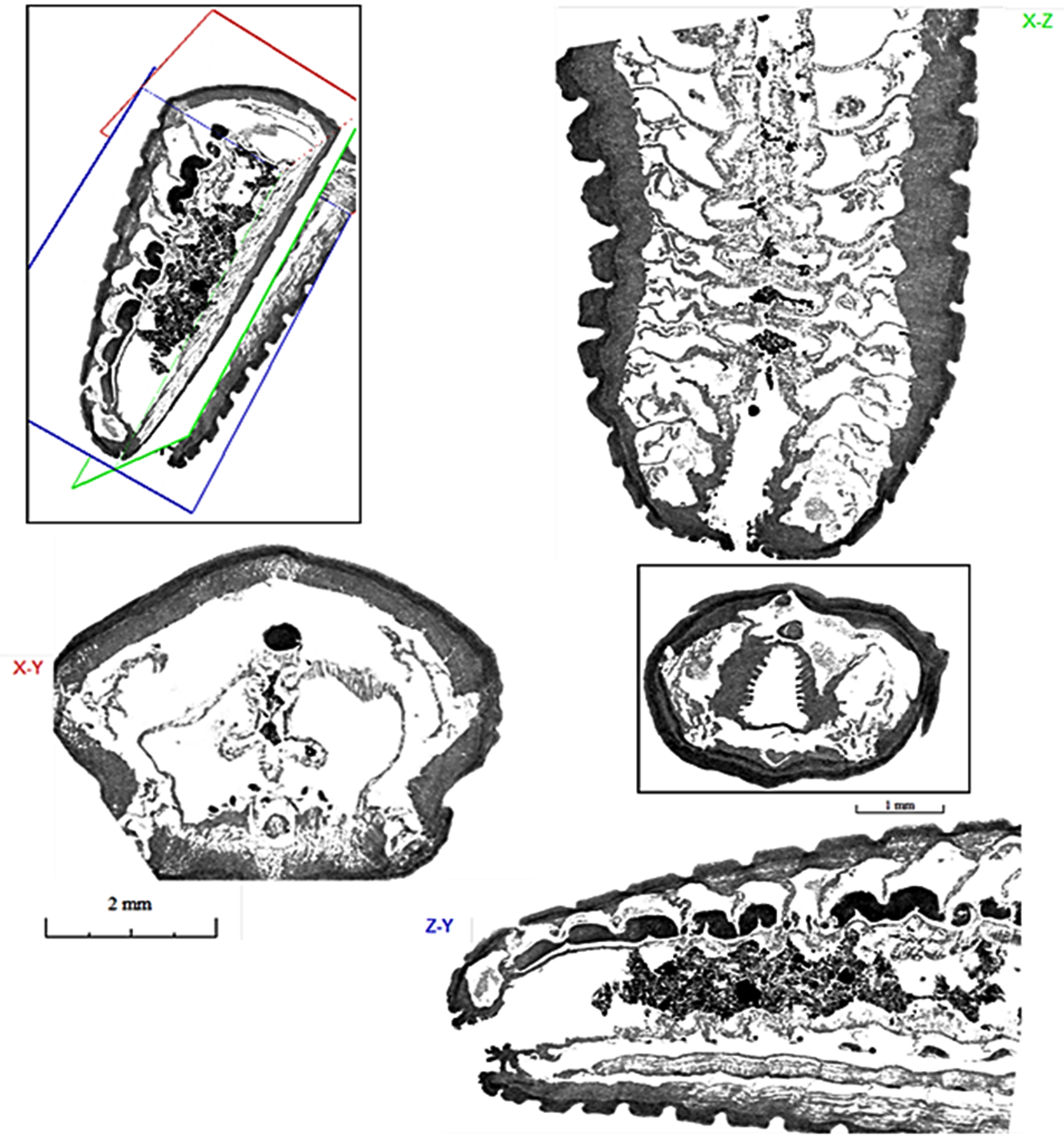


b)


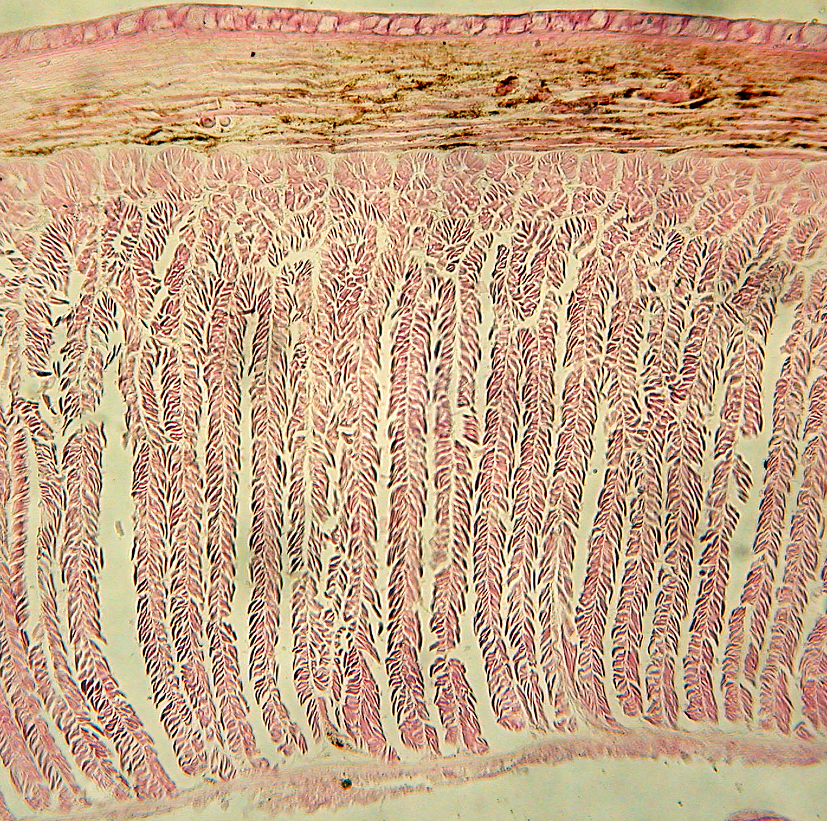


c)

Supplement: S3 Fig — a)7.98 µm on ventral, dorsal and lateral views and 3.08µm from Crevada (Crevada 6); b)virtual sections of hindmost segments: schematic and transversal sections of last ten segments where no typhlosole occurs; hindmost body segment- (middle) mesial-middle section (upper right), and sagittal medial section (bottom); transversal sections at level of the penultimate segment (middle right); c)cross section of longitudinal pinnate musculature (HNHM 6899 specimen). (DOC) [file pone.0160218.s003.doc]
